# Functional Studies of Heading Date-Related Gene *TaPRR73*, a Paralog of *Ppd1* in Common Wheat

**DOI:** 10.3389/fpls.2016.00772

**Published:** 2016-06-01

**Authors:** Wenping Zhang, Guangyao Zhao, Lifeng Gao, Xiuying Kong, Zhiai Guo, Bihua Wu, Jizeng Jia

**Affiliations:** ^1^Triticease Research Institute, Sichuan Agricultural UniversityChengdu, China; ^2^National Key Facility of Crop Gene Resources and Genetic Improvement, Institute of Crop science, Chinese Academy of Agricultural SciencesBeijing, China

**Keywords:** *TaPRR73*, association analysis, heading date, transgenic gene, *Triticum aestivum*

## Abstract

Photoperiod response-related genes play a crucial role in duration of the plant growth. In this study, we focused on *TaPRR73*, a paralog of “Green Revolution” gene *Ppd1* (*TaPRR37*). We found that overexpression of the truncated *TaPRR73* form lacking part of the N-terminal PR domain in transgenic rice promoted heading under long day conditions. Association analysis in common wheat verified that *TaPRR73* was an important agronomic photoperiod response gene that significantly affected heading date and plant height; expression analysis proved that specific alleles of *TaPRR73-A1* had highly expressed levels in earlier heading lines; the distribution of haplotypes indicated that one of these alleles had been selected in breeding programs. Our results demonstrated that *TaPRR73* contributed to regulation of heading date in wheat and could be useful in wheat breeding and in broadening adaptation of the crop to new regions.

## Introduction

Photoperiod genes in plants affect the timing of transition from the vegetative to reproductive phases. Allelic variation of photoperiod response genes enables common or bread wheat to adapt to different day-lengths characteristic of different latitudes and thus become more widely cultivated. The pseudo-response regulator (*PRR*) gene family is highly conserved in protein structure. These members have an N-terminal PR (pseudoreceiver) domain and a C-terminal CCT (CONSTANS, CO-like, TOC1) domain (Makino et al., 2000; Wenkel et al., 2006). There are five members in each of *Arabidopsis thaliana* (*TOC1, PRR3, PRR5, PRR7*, and *PRR9*) (Matsushika et al., 2000) and rice (*OsPRR1, OsPRR37, OsPRR73, OsPRR59*, and *OsPRR95*) (Murakami et al., 2003). Study of these family members in wheat are less reported, as most attention focused on *Ppd* (*TaPRR37*) (Beales et al., 2007; Guo et al., 2010). There has been one expression study of *TaPRR73* (Shaw et al., 2012).

*PRR73* and *PRR37* are paralogous genes that exist in plant genomes (Higgins et al., 2010). Paralogous genes make up a significant proportion of plant genomes, for example 22% of the rice genome (Goff et al., 2002), 50% in modern maize (Schnable et al., 2011), and more than 67% in soybean (Schmutz et al., 2010). Paralogous genes are derived from duplication events that occurred in the ancestors of modern species (Fitch, 1970; Van de Peer et al., 2009), and their functions may duplicate, or be differentiated from, those of their progenitors. Therefore, mining of the functions of paralogous gene series may have significance for both genetic analysis and breeding.

Numerous studies have demonstrated that *OsPRR37* plays important roles in increasing photoperiod sensitivity in rice. *OsPRR37* delays heading by repressing *Hd3a* under long day conditions (Koo et al., 2013). *TaPRR37 (Ppd)*, one of the well-known “Green Revolution” genes in wheat, is an important photoperiod gene associated with multiple agronomic traits. *Ppd-A1, Ppd-B1*, and *Ppd-D1*, located in the three sub-genomes, are orthologous photoperiod response gene loci, with the *a* alleles causing early flowering under both short and long photoperiods (Beales et al., 2007; Wilhelm et al., 2009; Bentley et al., 2011) and the *b* alleles conferring day length sensitivity that delays flowering under SD conditions (Laurie, 1997; Shaw et al., 2012). The *Ppd-D1* gene consists of six haplotypes and affects heading time, plant height and 1000-kernel weight (Guo et al., 2010). Multiple copies and/or higher methylation of *Ppd-B1* enhance expression levels and promote heading and photoperiod insensitivity (Díaz et al., 2012; Sun et al., 2014). In addition to their effects on flowering *Ppd* genes regulate inflorescence architecture and paired spikelet behavior (Boden et al., 2015), and may improve grain yield and seed threshability during harvesting (Doebley et al., 2006).

As a paralog of *PRR37, PRR73* may also have a potential role in regulation of flowering (Higgins et al., 2010; Shaw et al., 2012). Here, we analyzed the functions of *TaPRR73* in wheat by a transgenic approach, expression analysis, linkage mapping, and association analysis. Our results shed light on the potential value of *TaPRR73* in genetic improvement of cereals such as wheat, rice and barley.

## Materials and methods

### Plant material

Eleven hexaploid wheat accessions (Chinese Spring, Neixiang 188, Yanzhan 1, Opata M85, W7984, Am3, Am6, Laizhou 953, Fuzhang 30, Hanxuan 10, and Lumai 14) and 6 diploid accessions (UR201, UR203, UR206, A_BD104_, A_B08_, A_M0102_) were used for sequencing. Two hundred and seventy introgression lines (ILs) were derived from crosses of 30 donor varieties to Yanzhan 1, followed by four or five backcrosses to Yanzhan 1, and then selfed without selection for more than three generations. One hundred and fifty-six wild species are listed in Supplementary Tables 1, 2. Three hundred and eighty accessions (including landrace and modern cultivars listed in Supplementary Table 3) from 10 major wheat-growing regions of China were used in determining haplotype distributions. These were planted at Changping in Beijing (116.2°E, 40.2°N), and Luoyang (111.6°E, 33.8°N), Xinxiang (113.8°E, 35.2°N) and Jiaozuo (113.4°E, 35.10°N) in Henan province during years 2011–2014. A recombinant inbred line (RIL) population derived from cross Neixiang 188 × Yanzhan 1 (199 lines) was used for genetic mapping. Transgenic rice lines were planted at Langfang in Heibei province under long day conditions. All materials were provided by the Key Laboratory of Crop Gene Resources and Germplasm Enhancement, Institute of Crop Sciences, CAAS. Genomic DNA was extracted from all materials by a modified CTAB method (Saghai-Maroof et al., 1984).

### Phylogenetic analysis

The sequences of *TaPRR1, TaPRR59*, and *TaPRR95* were obtained from D genome scaffolds, and their protein constructs were predicted by PROSITE (http://www.expasy.ch/prosite/). Mega 5.0 software was used to produce a phylogenetic tree (http://www.megasoftware.net).

### Software analysis

Cis-regulatory elements were predicted by PLACE (Higo et al., 1999). Statistical analyses were conducted with SPSS 15.0 (SPSS Inc. Chicago, IL, USA) and Power Marker V3.25 (Liu and Muse, 2005).

### Primer design and PCR

Primers for amplifying the *TaPRR73* gene included the A genome-specific primer TaPRR73AF1/TaPRR73AR1 and B and D genome primers TaPRR73BDF1/TaPRR73BDR1, TaPRR73AF1: GCACCACCACTTCTCTCCTC; and TaPRR73AR1: CTACTGGCTTGCTCCTTCTT; TaPRR73BDF1: AAACGAGGACAAGGAATGGAGG; and TaPRR73BDR1: GGGACAATAATCATACGGGTGG.

RT-qPCR primers used for wheat were TaPRR73-A1F/TaPRR73-A1R, TaPRR73-B1F/TaPRR73-B1R, TaPRR73-D1F/TaPRR73-D1 (Shaw et al., 2012) and TaPRR73-F/PRR73-R; and primer sets OsHd1-F/OsHd1-R (Kojima et al., 2002), OsGI-F/OsGI-R and OsMADS51-F2/OsMADS51-R2 were used in transgenic rice (OsGI-F2: CCGAATACTCTCCCAACCGA and OsGI-R2: AAACCATACGCAGCCTCCCA; OsMADS51-F2: GTCTCTCCAAAACAATGC; and OsMADS51-R2: TCTGCTCCTACTCCCTTC). High-efficiency thermal asymmetric interlaced PCR (hiTAIL-PCR) was used to isolate T-DNA-flanking sequences from transgenic rice plants (Liu and Chen, 2007). All primers were synthesized by Sangon (www.sangon.com). LA-Taq enzyme from TaKaRa (www.takara.com.cn) was used for PCR amplification, and Pfu was included at 1/10th of the total enzyme concentration to ensure amplification accuracy. The PCR mixture comprised 5 μL of 2 × GC buffer, 2.5 μL ddH_2_O, 1.5 μL DNA (20 ng/μL) or cDNA as template, 0.4 μL of each primer (10 μmol/L), 0.1 μL dNTP (25 mmol/L), and 0.1 μL LA-Taq (5 U/μL) in a total volume 10 μL. The PCR protocol was 95°C for 5 min; 95°C for 40 s, primer annealing at 58°C for 40 s, and extension at 72°C for 1 Kb/min for 32 cycles and a final extension at 72°C for 10 min.

### Marker development

Marker PASF2/PASR2 was developed based on the 9 bp indel difference between Hap 1 and Hap 2 of *TaPRR73-A1* (PASF2: TTTGTAGTTATCGCTGCTGAGAA; PASR2: AAC AAGGACCAAAATAAGCGTAT). Marker URSF1/URSR1 was designed according to the 10 bp indel present in the third exon of diploid lines (URSF1: ACGGGTGGGTCTTTATTTGTT; URSR1: GCCTCATCTGCTTGGCTATTT). Marker 73ASF1/73ASR1 was designed according to a 306 bp indel differentiating hexaploid (Hap 1 and Hap 2) and diploid (Hap 3 and Hap IV) haplotypes (73ASF1: GTCGTTTGTCAACCGTCTCT; 73ASR1: CAGGGCATTACCTTCATAGC). Allele-specific markers (CF4/R4; TF4/R4) to distinguish *TaPRR73-B1* haplotypes were designed according to SNPs in the two haplotypes (CF4: ATG ACTGTACCCGACATATC; TF4: ATGACTGTACCCGACATGCT; R4: CAGCCAACCATTGCATGCA).

### Transgenic vector construction

We firstly constructed a binary vector by combing the pCUbi1390 vector and Gateway cassete A including the maize ubiquitin promoter, NOS terminator, and hygromycin resistance. We inserted the truncated cDNA of TaPRR73 partially lacking the N-terminal PR domain into the binding vector by Gateway technology (Life technologies, Invitrogen), and then transformed it into japonica rice cultivar (cv.) Nipponbare.

### Expression analysis

Wheat accession Hussar and rice accession Nipponbare and transgenic lines were planted under long (15 h light, 9 h darkness) and short (9 h light, 15 h darkness) day conditions. Each treatment was sampled every 3 h during a 48 h period; at each time-point samples from three plants were pooled. Plant organs including roots, shoots, leaves, and young ears were taken from one plant of Chinese Spring under natural long day conditions (LDs). Total mRNA was extracted with an RNA extraction kit (Tiangen Biotech, Beijing) and reverse transcribed with Moloney Murine Leukemia Virus (M-MLV) (Invitrogen). Real-time qPCR (RT-qPCR) was performed on an ABI PRISM 7900 (Applied Biosystems, USA) with SYBR Premix ExTaqII (Takara), and data were quantified by the 2^−ΔΔ*Ct*^ method (Livak and Schmittgen, 2001). Wheat and transgenic rice expression data were normalized by GADPH and tubulin, respectively. Microarray data for *TaPRR73, Ppd, HvPRR73, OsPRR73*, and *OsPRR37* were obtained from the Genevestigator database (https://www.genevestigator.com/gv/) (Zimmermann et al., 2004). Expression patterns of these genes were compared after all data were standardized by the Z-score method (Benedito et al., 2008). A heatmap was drawn by MeV4.9 (http://www.tm4.org/mev.html).

## Results

### Overexpression of a truncated *TaPRR73* promoted heading in rice under long day conditions by suppressing expression of *OsGI*

**Figure 1:**
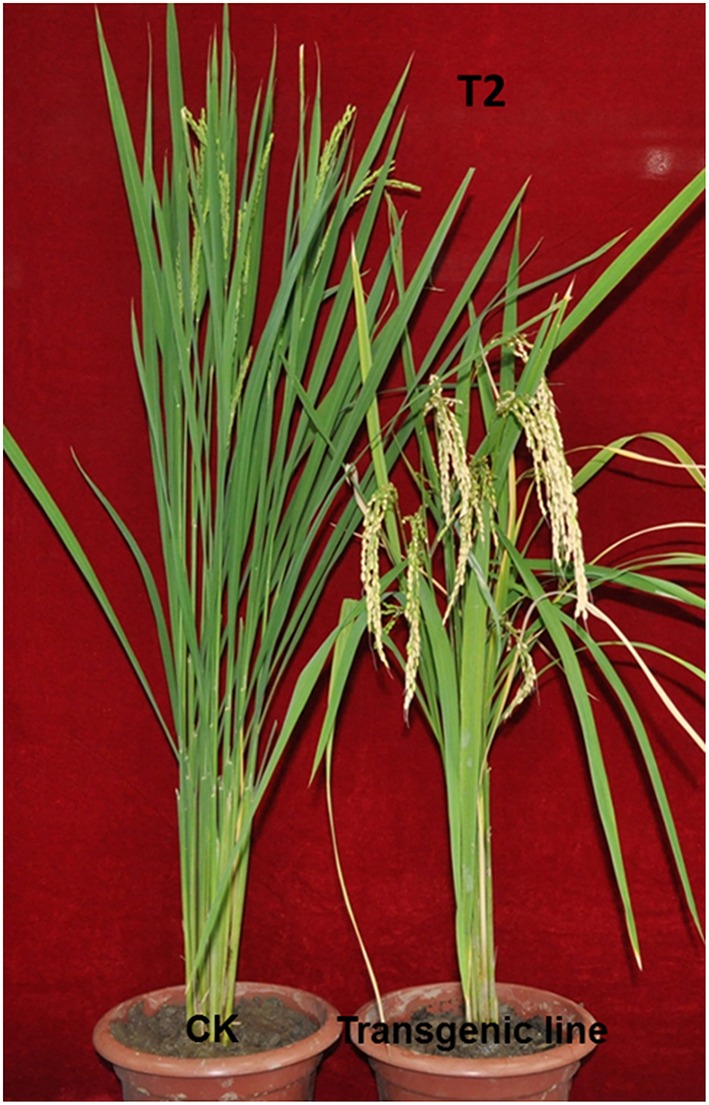
**Comparison of T_**2**_ transgenic (right) and control (left) plants (cv. Nipponbare)**.

To explore the role of *TaPRR73* in heading, we constructed a truncated *TaPRR73* expression vector, beginning with the second ATG in the intact gene sequence (Supplementary sequence 1), and transformed it into cv. Nipponbare. The sequence was based on a platform of wheat genes transformed into rice to mine new functional genes. Five lines including the truncated *TaPRR73* were obtained, but three of them contained other inserted genes. Two independent T_0_ transgenic lines were 6 days earlier in heading than the controls under long day conditions (LDs). Next we determined the transgene position in the rice genome by hiTAIL-PCR (Liu and Chen, 2007) and found that it was inserted into a retrotransposon rather than a promoter or coding region. T_1_ generation transgenic lines planted in natural LDs were significantly earlier heading (113.7 days) than wild type (119.9 days) (*p* < 0.01) (Table 1). T_2_ generation plants were even earlier heading (nearly 15 days) than the Nipponbare control (Figure 1).

**Table 1:** *****T***-test of days to heading of truncated ***TaPRR73-B1*** T1 transgenic and nontransgenic rice plants**.

	**Number of lines**	**Mean (days) ± SD**	***t***	***p***
Transgenic lines	20	113.7 ± 2.98	4.868	0.000^**^
Non-transgenic control	8	119.9 ± 3.18		

** *Significantly different at p = 0.01*.

**Figure 2:**
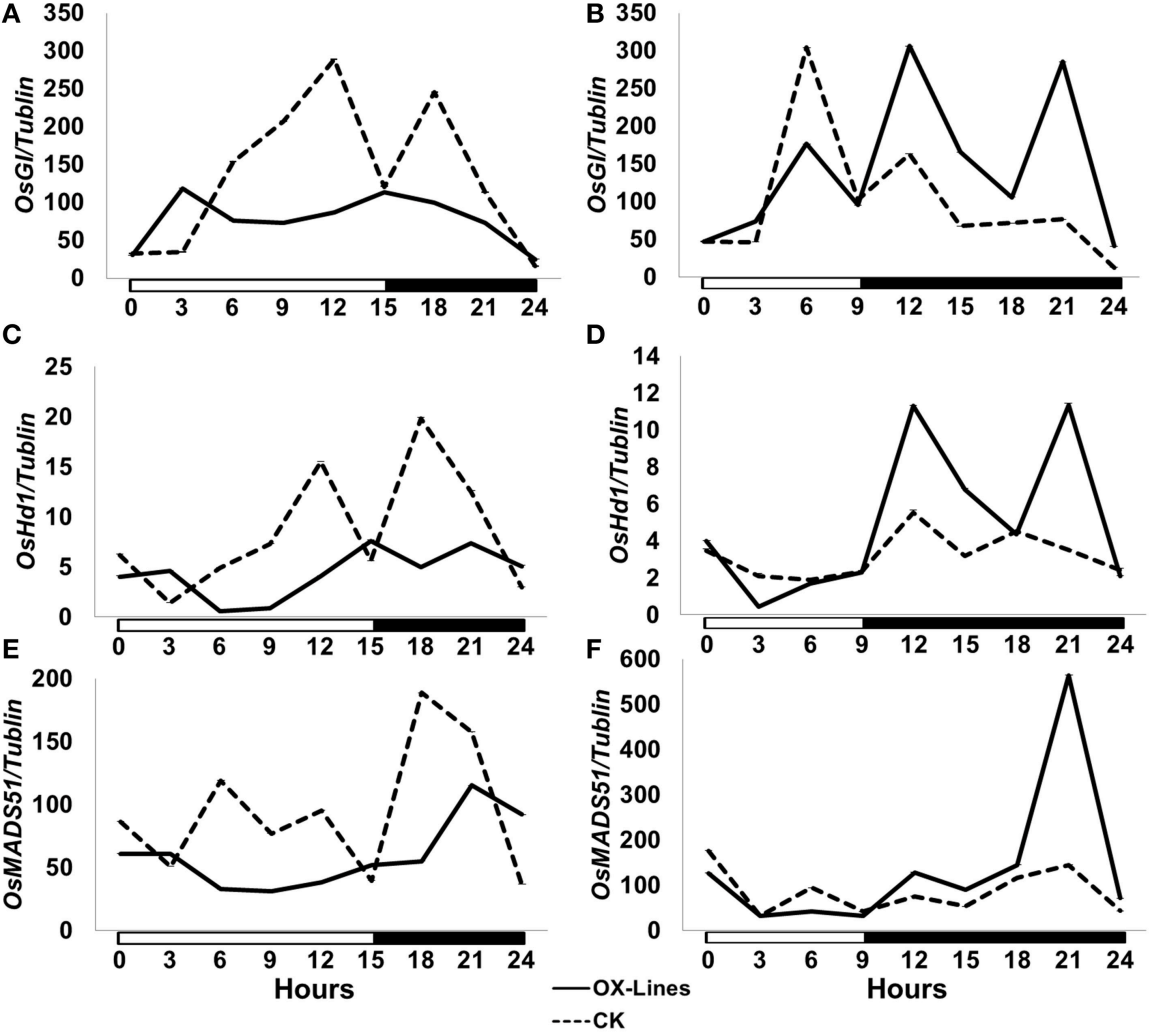
**Expression of major photoperiod genes in truncated ***TaPRR73-OX*** rice lines and cv. Nipponpare check. (A,C,E)** Expression levels of *OsHd1, OsGI*, and *OsMADS51* under LDs (15 h light/ 9 h darkness). **(B,D,F)** The expression level of *OsGI, OsHd1*, and *OsMADS51* under SDs (9 h light/ 15 h darkness). Four biologically independent replications were performed for each sample. White boxes below the graphs indicate light periods; dark boxes indicate darkness. The data were normalized by tubulin.

To investigate the mechanism of action of *TaPRR73* in rice we compared the expression patterns of heading date-related genes *OsGI, OsHd1*, and *OsMADS51* in transgenic lines under LDs and SDs (Figure 2). Under LDs, *OsGI* and *OsHd1* suppress flowering in rice (Yano et al., 2000; Hayama et al., 2003) whereas expression of the genes in our transgenic lines was suppressed, suggesting that truncated *TaPRR73* promoted heading by inhibiting expression of heading date suppressors under LDs. Under SDs, *OsGI* suppresses flowering (Hayama et al., 2003) whereas *OsHd1* and *OsMADS51* promote heading in rice (Yano et al., 2000; Kim et al., 2007). Expression levels of *OsGI, OsHd1*, and *OsMADS51* in the transgenic lines increased during darkness.

### The *PRR* gene family members have a conserved protein structure and might be similar in function

**Figure 3:**
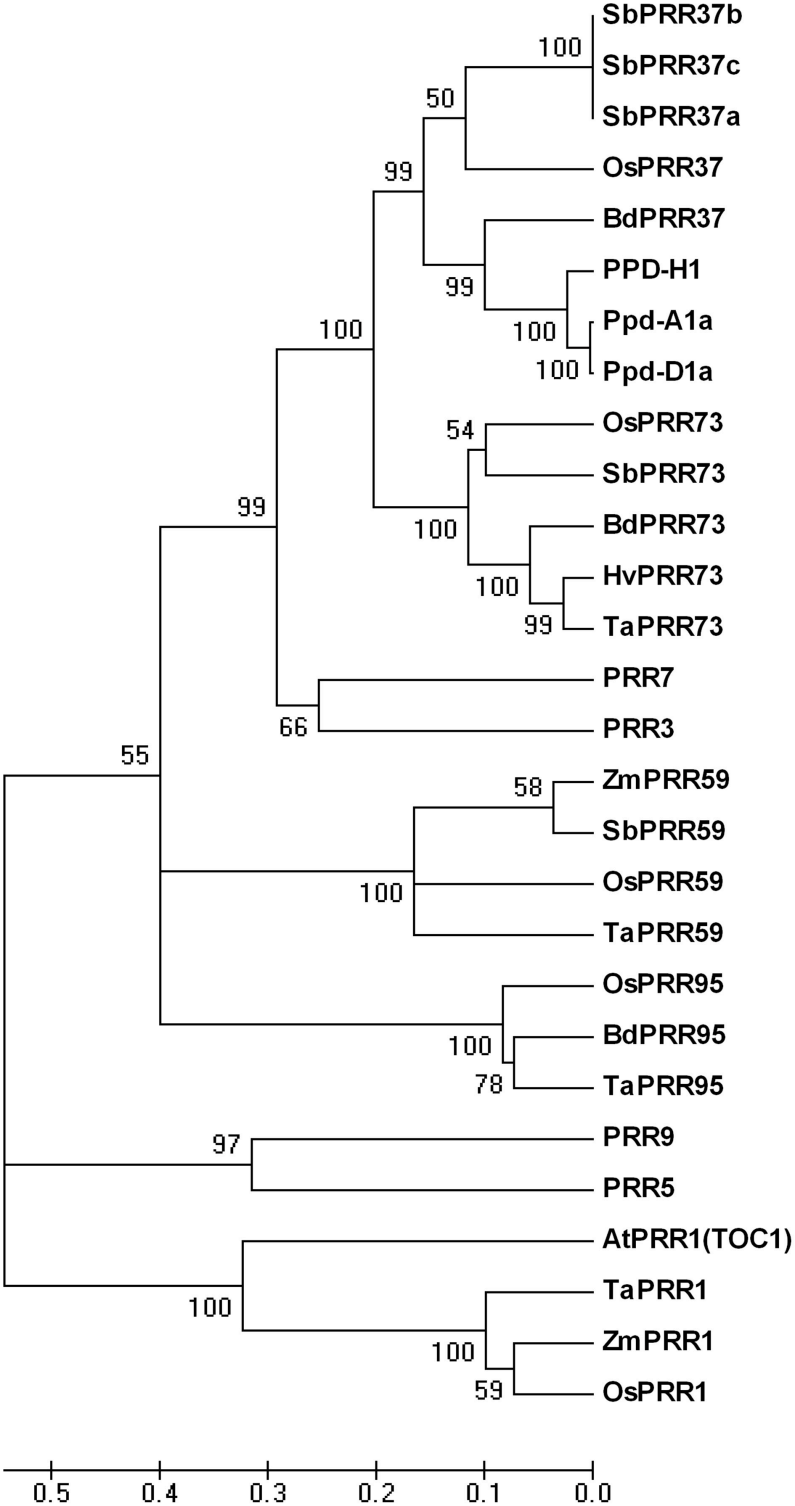
**Phylogenetic tree of PRR proteins from ***Triticum aestivum***, ***Oryza sativa***, ***Hordeum vulgare***, ***Sorghum bicolor***, ***Brachypodium distachyon***, ***Zea mays***, and ***Arabidopsis thaliana*****. Accession no. of *Oryza sativa, Hordeum vulgare, Sorghum bicolor, Brachypodium distachyon, Zea mays*, and *A. thaliana* proteins were obtained from NCBI; *Triticum aestivum* Ppd-D1a, ABL09464; and Ppd-A1a, ABW93666. The amino acid sequences of *TaPRR1, TaPRR59*, and *TaPRR95* were predicted from the D genome scaffold.

To study variation and function of *PRR73* in common wheat and compare it with other *PRR* families, we isolated *TaPRR73-A1, TaPRR73-B1*, and *TaPRR73-D1* in Chinese Spring according to the cDNA sequence in transgenic lines and D genome scaffolds (Jia et al., 2013), and also compared the amino acid sequences of family members in *Triticum aestivum, Oryza sativa, Arabidopsis thaliana, Hordeum vulgare, Sorghum bicolor, Brachypodium distachyon*, and *Zea mays* by constructing a genetic phylogenic tree. The phylogenic tree divided into three clusters, revealing that the PRRs were very similar in monocots *Triticum aestivum, Oryza sativa, Hordeum vulgare, Sorghum bicolor, Brachypodium distachyon*, and *Zea mays* (Figure 3). PRR73 and PRR37 were highly similar in monocots and closest to PRR3 and PRR7 in the dicotyledon *A. thaliana*. PRR59 and PRR95 were also similar in monocots and orthologous with PRR9 and PRR5 in *A. thaliana*. The PRR1 cluster had a similar pattern. However, the PRR1 cluster had least similarity with the other clusters, indicating a difference in amino acid sequence and possibly in function. Research on *PRR* gene function has shown that *PRR37* and *PRR73* were morning-expressed circadian genes, whereas *TOC1* was evening-expressed (Higgins et al., 2010). This suggested that *PRR37* and *PRR73* might have different ways of regulating photoperiod response compared to *TOC1*. All the above results demonstrated that PRRs are highly conserved in monocots.

### *TaPRR73* exhibited a circadian rhythm and higher expression level in leaf tissue

**Figure 4:**
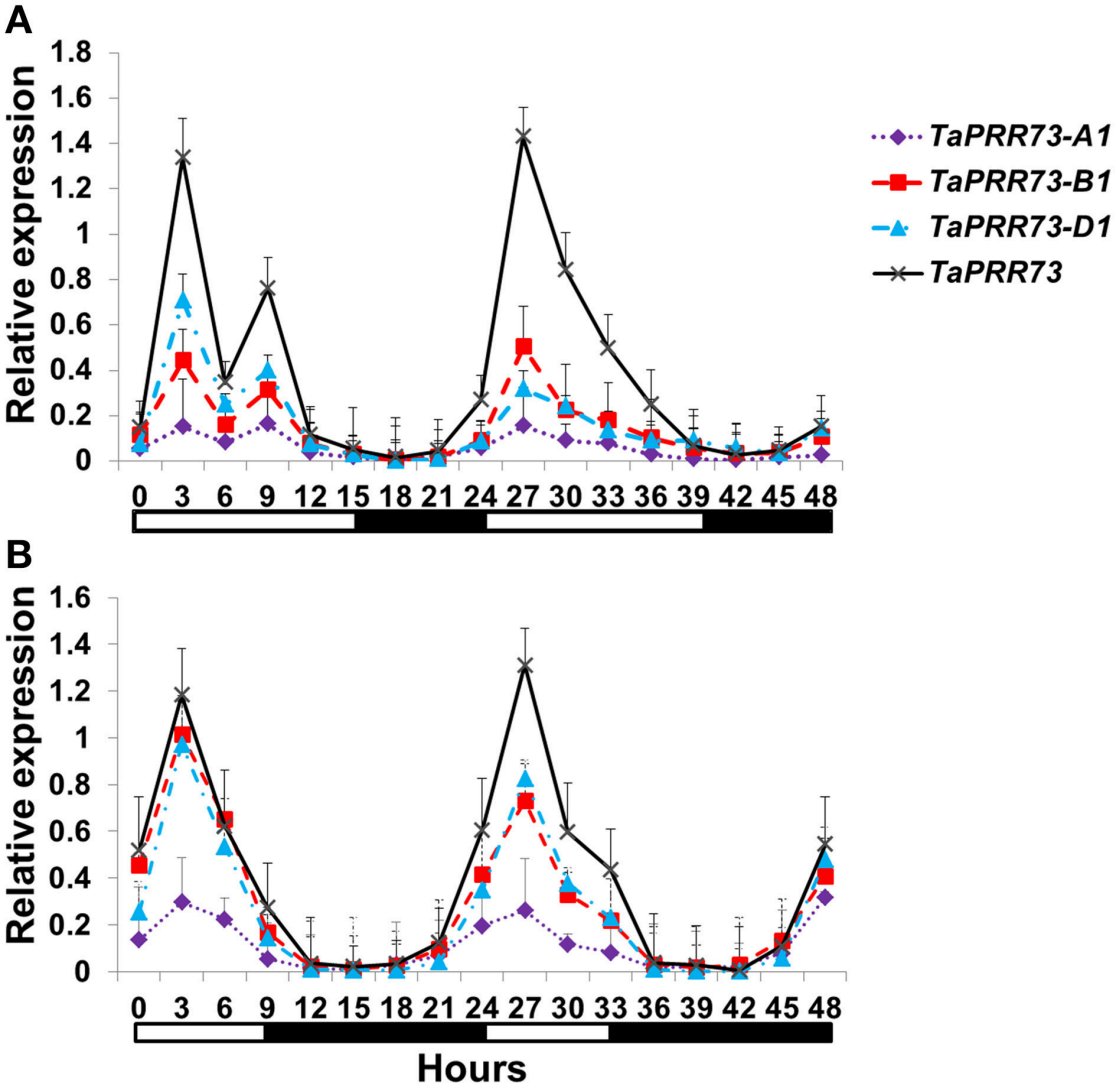
**Relative circadian expression of ***TaPRR73*** in cv. Hussar under LD and SD conditions. (A)** *TaPRR73, TaPRR73-A1, TaPRR73-B1*, and *TaPRR73-D1* in LDs. **(B)** *TaPRR73, TaPRR73-A1, TaPRR73-B1*, and *TaPRR73-D1* in SDs. The expression level of *TaPRR73* reached a peak 3 h after dawn. The contrasting environments were 15 h light/9 h darkness and 9 h light/15 h darkness. Four biologically independent replications were performed for each sample. White boxes below the graphs indicate light periods; dark boxes indicate darkness. The data were normalized by GADPH.

RT–qPCR was used to investigate the expression patterns of *TaPRR73* during a 48 h period in common wheat cv. Hussar grown under SD and LD conditions. *TaPRR73* was expressed mainly during the light period under both LD and SD conditions, and its transcript levels peaked 3 h after dawn (Figure 4) as reported in previous studies (Wilhelm et al., 2009; Shaw et al., 2012). *OsPRR73* and *OsPRR37* in rice also had the same expression peak, and five *PRR*s expressed in a sequential manner of *OsPRR73* (*OsPRR37*) → *OsPRR95* (*OsPRR59*) → *OsPRR1* (Murakami et al., 2005). In *Arabidopsis*, five members expressed in the order *AtPRR9, AtPRR7, AtPRR5, AtPRR3*, and *TOC1* over a 24 h period (Matsushika et al., 2000). From our results and published data we concluded that the paralogous gene pairs *TaPRR73* and *TaPRR37*, and *OsPRR73* and *OsPRR37* are orthologs of *AtPRR7* and behave in a similar circadian manner.

**Figure 5:**
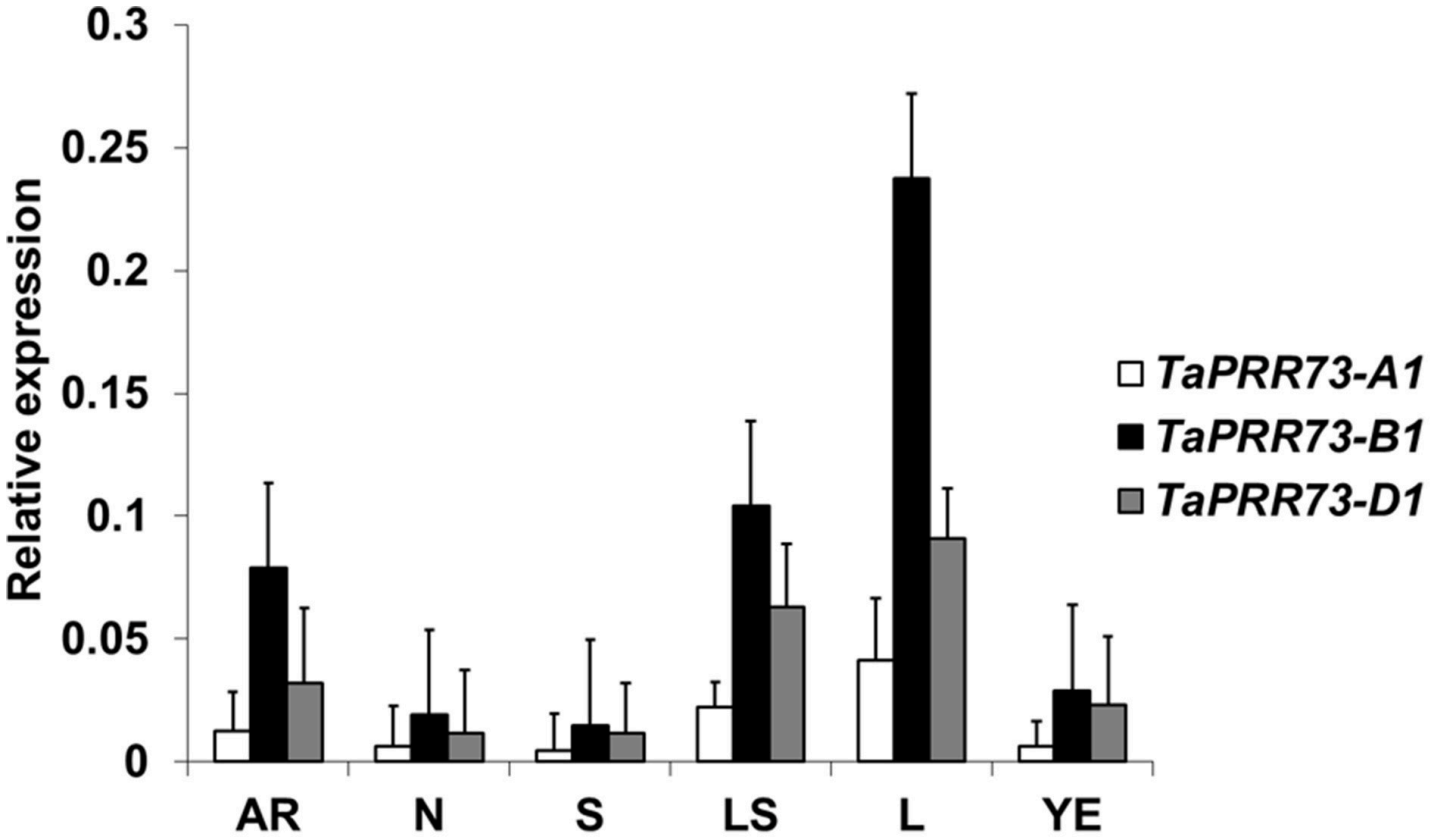
**Expression levels of the three ***TaPRR73*** homologs in different organs**. AR, adult roots; N, nodes; S, shoots; LS, leaf sheaths; L, leaves; YE, young ears. Ranking of expression levels from highest to lowest was L > LS > AR > YE > N > S.

We investigated expression levels of the three orthologous *TaPRR73* genes in six different organs of Chinese Spring (Figure 5), collected from 9 to 10 am during the day. Expression levels of the three homoeologs ranked *TaPRR73-B1* > *TaPRR73-D1* > *TaPRR73-A1* (Figure 5). All three were more highly expressed in leaves than in other organs, with expression levels from highest to lowest being leaves > leaf sheaths > adult roots > young ears > nodes > shoots, in accordance with *in-silico* expression data (the Genevestigator database) (Supplementary Figure 1). Both *TaPRR73* and *Ppd* had the highest expression levels in leaves and lowest expression levels in endosperm as determined from microarray data for *OsPRR73, OsPRR37*, and *Ppd1* (*TaPRR37*) (Genevestigator database). However, *TaPRR73* also had high expression levels in roots and pistils, whereas the corresponding expression levels of *Ppd* in roots and pistils were lower than in other organs, suggesting partial differentiation in function of the two genes.

To investigate expression differences between *TaPRR73* and *Ppd1* (*TaPRR37*), we compared their coding and promoter regions. They shared high similarity in amino acid sequence (68.2%), especially in the CCT domain region (95.2%), but had large differences in the promoter regions. We analyzed the cis-regulatory elements in 2000 bp segments upstream of the ATG start codons of *TaPRR73-B1* and *Ppd1* (*TaPRR37-B1*). Although, both paralogs had a light-responsive element, a hormone-responsive element, and abiotic-responsive elements, the element sequences were different and bound different proteins. Moreover, there were a root hair-specific cis-element and a dehydration-responsive element in *TaPRR73-B1*, suggesting specific effects on roots (Supplementary Figure 2). The different cis-regulatory elements in the promoter may cause functional differentiation of these paralogous genes.

### Haplotype variation and linkage analysis of *TaPRR73*

In order to detect variation in *TaPRR73-A1, TaPRR73-B1*, and *TaPRR73-D1*, we sequenced the coding and promoter regions in four diploid accessions and 11 hexaploid accessions. There were four haplotypes of *TaPRR73-A1* and two haplotypes of *TaPRR73-B1*, but no variation in *TaPRR73-D1* in the tested hexaploid wheat accessions.

**Figure 6:**
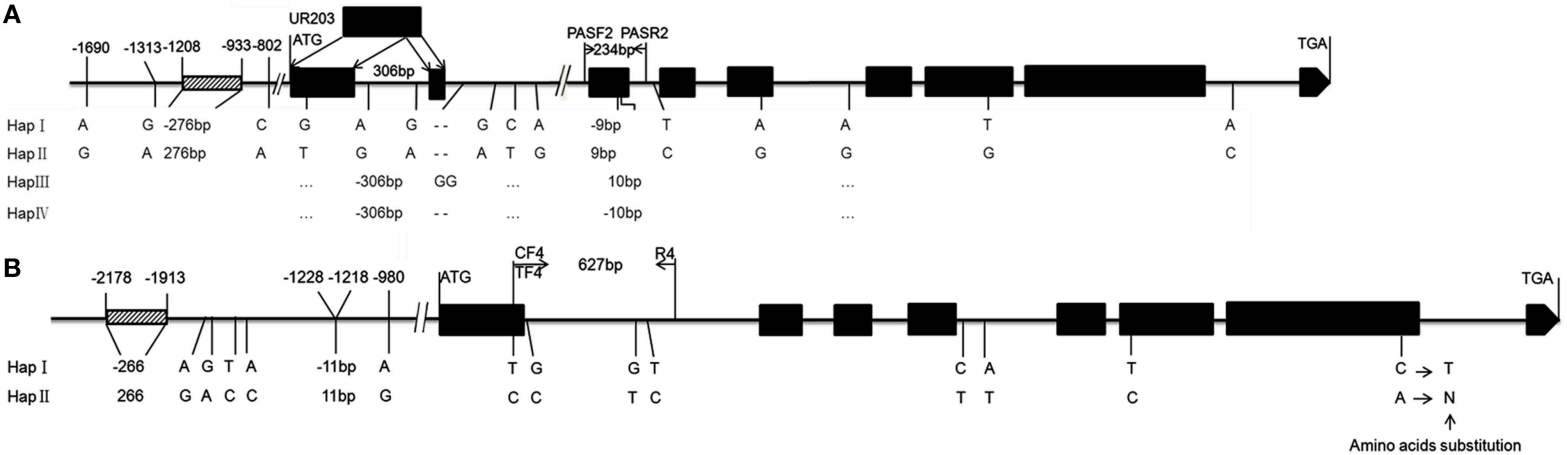
**Gene structures of ***TaPRR73-A1*** and ***TaPRR73-B1*****. Structure and SNPs and indels in the four *TaPRR73-A1* **(A)** and two *TaPRR73-B1* **(B)** haplotypes are shown below the diagrams.

We compared *TaPRR73-A1* haplotypes in common wheat (HapI and HapII) and wild species (Hap3 and Hap4) (Supplementary Figures 3A–C). A 306 bp insertion in common wheat led to an additional exon in *TaPRR73-A1* compared *TaPRR73-B1* (Figure 6A). Three SNPs and a 276 bp indel in the promoter region, and 12 SNPs and a 9 bp indel in coding region in *TaPRR73-A1* formed two haplotypes. The twelve SNPs in the coding region caused no amino acid substitutions, but the 9 bp indel in the third exon resulted in a 3 amino acid indel (GIG) that potentially could lead to functional polymorphism. In *TaPRR73-B1* 13 SNPs and 2 Indels resulted in two haplotypes (Figure 6B, Supplementary Figure 3E). Differences included two indels (206 and 11 bp) and five SNPs in the promoter region, and eight SNPs in the coding region. The SNPs in the first and sixth exons caused no amino acid substitutions; the SNP in the seventh exon led to an amino acid substitution: asparagine (N) at position 681 in Hap 1 to threonine (T) in Hap II.

To map *TaPRR73*, marker PASF2/PASR2 based on the 9 bp indel was developed to distinguish *TaPRR73-A1* HapI and HapII. This marker was then utilized to map *TaPRR73-A1* in chromosome 4A in the recombinant inbred line (RIL) population developed from the cross Neixiang 188 × Yanzhan 1. *TaPRRR73* was located between markers WMC516 and W7001, with genetic distances of 8.6 and 6.2 cM, respectively (Supplementary Figure 3D).

### *TaPRR73* is associated with heading date and plant height

The Yanzhan 1 introgression lines were used to further examine the relationship between *TaPRR73* and agronomic traits. *TaPRR73-A1* was significantly associated with heading date (phenotypic variation explained (PVE) ranging from 2.48 to 17.09%) and plant height (PVE, 2.98–7.55%). HapI accessions were earlier heading (0.6–3 days) and taller (4.31–6.44 cm) than HapII accessions under long day conditions (Table 2). *TaPRR73-B1* was also significantly associated with heading date (PVE ranging from 2.26 to 4.39%), and plant height (PVE, 2.7–8.08%). Hap II was earlier heading (0.7–2.2 days), and taller (5.11–8.19 cm) than Hap I (Table 3).

**Table 2:** **Association analysis of two ***TaPRR73-A1*** haplotypes in different environments**.

**Trait**	**Year**	**Environment**	**Number of accessions**		**Mean (days)** ± **SD**		***p***	**PVE (%)**
			**Hap I**	**Hap II**	**Hap I**	**Hap II**		
Days to heading	2011	Xinxiang	146	37	186.5 ± 3.2	189.5 ± 3.8	0.000^**^	17.09
	2013	Xinxiang	206	52	185.0 ± 3.6	186.7 ± 3.9	0.003^**^	4.77
		Shunyi	157	47	217.0 ± 1.8	217.6 ± 1.4	0.045^*^	2.48
	2014	Xinxiang	141	41	175.4 ± 4.1	178.0 ± 3.8	0.000^**^	8.65
		Shunyi	130	40	204.5 ± 3.1	205.8 ± 2.9	0.024^*^	3.74
		Jiaozuo	141	41	170.6 ± 3.3	173.1 ± 3.5	0.000^**^	11.37
Plant height (cm)	2012	Xinxiang	204	52	73.97 ± 10.74	69.85 ± 13.49	0.045^*^	2.98
	2013	Xinxiang	206	52	78.55 ± 10.82	72.11 ± 11.58	0.000^**^	7.55
		Shunyi	157	47	68.76 ± 10.02	64.36 ± 11.56	0.021^*^	3.92
	2014	Xinxiang	141	41	81.16 ± 12.96	75.72 ± 14.24	0.022^*^	3.72
		Shunyi	130	39	69.07 ± 11.47	64.76 ± 12.40	0.045^*^	2.99
		Jiaozuo	141	41	69.74 ± 10.99	65.13 ± 10.72	0.018^*^	3.92

Significantly different at

* *P = 0.05*,

** *P = 0.01*.

**Table 3:** **Association analysis of two ***TaPRR73-B1*** haplotypes in different environments**.

**Trait**	**Year**	**Environment**	**No. of accessions**		**Mean** ± **SD**		***p***	**PVE (%)**
			**Hap I**	**Hap II**	**Hap I**	**Hap II**		
Days to heading	2011	Xinxiang	36	153	188.5 ± 3.7	186.7 ± 3.4	0.007^**^	4.35
	2012	Xinxiang	66	204	184.6 ± 5.2	182.9 ± 3.3	0.003^**^	2.81
	2013	Xinxiang	65	201	186.7 ± 4.5	185.0 ± 3.2	0.005^**^	3.66
		Shunyi	55	155	217.6 ± 1.6	216.9 ± 1.8	0.015^*^	2.26
	2014	Xinxiang	52	138	177.5 ± 4.3	175.3 ± 3.9	0.001^**^	4.09
		Shunyi	49	129	205.8 ± 3.0	204.4 ± 3.1	0.006^**^	3.2
		Jiaozuo	52	138	172.4 ± 3.5	170.6 ± 3.3	0.001^**^	4.39
Plant Height (cm)	2012	Xinxiang	65	200	69.60 ± 14.99	74.71 ± 11.12	0.013^*^	2.7
	2013	Xinxiang	65	201	71.03 ± 12.85	79.22 ± 10.24	0.000^**^	8.08
		Shunyi	55	155	64.42 ± 14.25	69.11 ± 9.32	0.026^*^	2.81
	2014	Xinxiang	52	138	76.19 ± 16.15	81.89 ± 12.18	0.024^*^	2.67
		Jiaozuo	52	138	65.04 ± 12.48	70.51 ± 10.50	0.006^**^	3.53

Significantly different at

* *P = 0.05*,

** *P = 0.01*.

**Figure 7:**
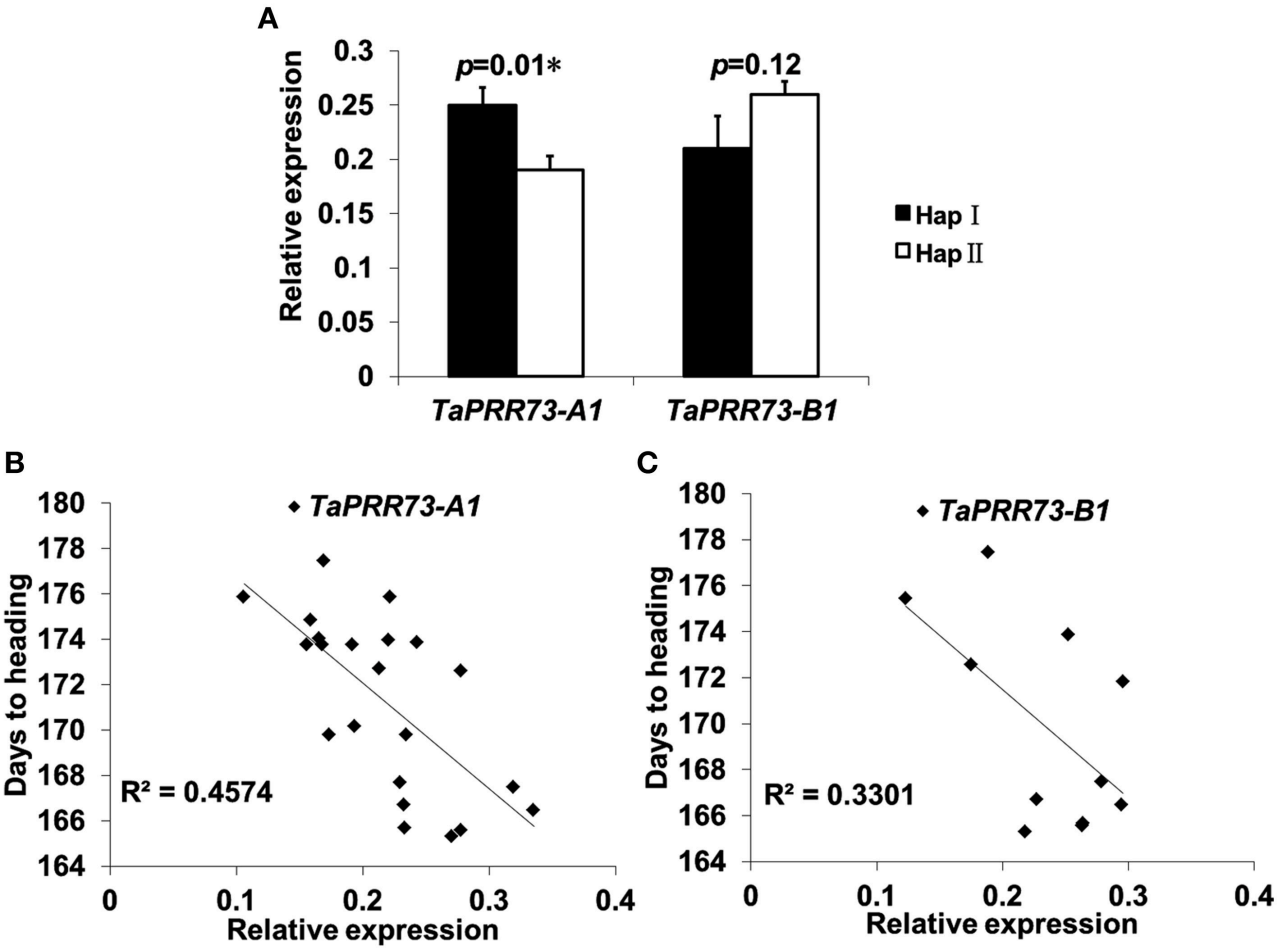
**Relative expression levels of different haplotypes and correlations between relative expression levels and days to heading. (A)** Relative peak time expression of *TaPRR73-A1* and *TaPRR73-B1* for different haplotypes grown under long day conditions. Three biologically independent replications were performed for each sample, and five or six accessions were tested for each haplotype. Standard deviations of means are indicated by error bars. Correlation analyses of relative expression levels and days to heading for *TaPRR73-A1* **(B)** and *TaPRR73-B1* **(C)**.

Because of the significant associations between haplotypes and traits we examined gene expression levels by RT-qPCR. Relative expression of *TaPRR73-A1* HapI (0.250) was higher than HapII (0.191) (*p* = 0.01), whereas relative expression of *TaPRR73-B1* HapII (0.258) was higher than HapI (0.207) (Figure 7A). There was a significant negative correlation between relative expression and days to heading in TaPRR73-A1 (*R*^2^ = 0.4574, *r* = −0.676, *p* < 0.01; Figure 7B), but not in TaPRR73-B1 (*R*^2^ = 0.5501, *r* = −0.575, *p* > 0.05; Figure 7C). Higher expression levels of *TaPRR73-A1* HapI led to earlier heading.

### Haplotype analyses of *TaPRR73-A1* and *TaPRR73-B1*

**Figure 8:**
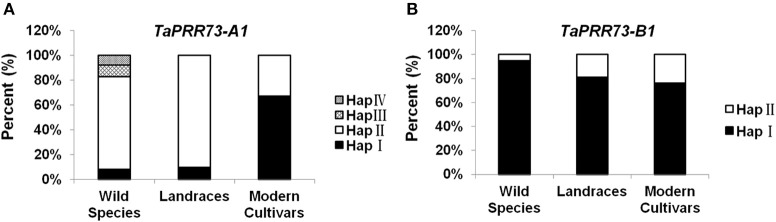
**Distributions of haplotypes of ***TaPRR73-A1*** (A) and ***TaPRR73-B1*** (B) in wild species, landraces, and modern cultivars**.

Allele frequency is an indicator of past selection in breeding. *TaPRR73-A1* Hap I (7.69%), Hap II (75%), Hap III (9.62%), and Hap IV (7.69%) were detected in wild species, but Hap III and Hap IV were absent in common wheat landraces and modern cultivars. Hap II was the dominant haplotype in wild species (46.34%) and landraces (86.52%), but its frequency was lower (33.06%) in modern cultivars. Hap I was present in 6.10 and 13.48% of wild species accessions and landraces, and 66.94% in modern cultivars (Figure 8A). We analyzed haplotype frequencies of *TaPRR73-A1* by PowerMarker V3.25 (Liu and Muse, 2005), and found that the frequencies for *TaPRR73-A1* differed significantly between landraces and modern cultivars (*u* = 7.99 > 3.29; *p* < 0.001), suggesting that Hap I was a favored haplotype selected in modern breeding programs. When the frequencies of the two *TaPRR73-B1* haplotypes were tested in the same way (*u* = 0.49, *p* > 0.05) there was no difference between landraces and modern cultivars and hence no evidence of previous selection for either haplotype (Figure 8B).

## Discussion

### Overexpression of truncated *TaPRR73* accelerates heading by regulating expression of *OsGI* in transgenic rice under LDs

Gene expression analyses showed that overexpression of truncated *TaPRR73* promotes heading in transgenic rice by reducing expression of *OsGI, OsHd1*, and *OsMADS51* under LDs. *OsHd1* and *OsMADS51* are downstream of *OsGI* in the rice flowering pathway (Hayama et al., 2003; Kim et al., 2007). *OsHd1* activates *OsHd3a* to promote flowering in SDs, and down-regulates *OsHd3a* to inhibit heading under LDs (Yano et al., 2000; Kim et al., 2007). *OsGI* is an ortholog of *GI* (Fowler et al., 1999) and suppresses flowering when overexpressed in transgenic rice, leading to late flowering under both SDs and LDs (Hayama et al., 2003). *OsGI* regulates expression of *OsHd1* and *OsMADS51*, and both *OsHd1* and *OsMADS51* promote heading under short-day conditions (Yano et al., 2000; Kim et al., 2007). In truncated *TaPRR73-OX* lines, expression of *OsGI* was repressed and transgenic plants flowered earlier under LDs. We therefore concluded that overexpression of truncated *TaPRR73* advances heading date by reducing the expression level of *OsGI* in transgenic rice under LDs.

Transgenic rice lines with the truncated *TaPRR73* exhibited earlier heading indicating that TaPRR73 may function as a regulator of heading in common wheat. However, we also raise the question of whether overexpression of truncated *TaPRR73* affects heading in transgenic rice plants by interfering with the function of the rice orthologs of TaPRR73 or acts independently. This will be addressed in future studies.

The intermediate region (IR) and CCT domains were present and involved no frame-shifts. The repression motif and CCT domain are important parts of PRRs in regulating downstream genes in Arabidopsis (Nakamichi et al., 2010; Gendron et al., 2012). *TOC1* is a critical circadian component of a feedback loop acting as a DNA-binding transcription repressor. It binds directly to the promoters of *CCA1*/*LHY* by its CCT domain to repress their expression (Gendron et al., 2012). The repression motif is in the pseudoreceiver (PR) domain of *TOC1*, and it alone cannot repress *CCA1* expression in the absence of the CCT and IR domains (Gendron et al., 2012). However, the repression motif is present in the IR between the PR and CCT domains of *PRR5, PRR7*, and *PRR9* (Nakamichi et al., 2010). Moreover, the CCT motif mutation (*toc1-2*) reduces the expression level of *CCA1*/*LHY* in *Arabidopsis* under LDs, whereas the PR domain mutation (*toc1-1*) continues to have some *TOC1* function (Millar et al., 1995; Strayer et al., 2000; Alabadí et al., 2001).

### *TaPRR73* is an agronomically important heading date gene in wheat breeding

*PRR37* (*Ppd1*) is an agronomically important photoperiod response gene that made a significant contribution in wheat, barley and rice breeding in the “Green Revolution”. In the present study, we found that *TaPRR73*, a paralog of *TaPRR37*, is also an important heading date-related gene lso affects plant height. The expression of *TaPRR73* in roots is relatively high, whereas there is negligible expression of *TaPRR37*. A comparison of *TaPRR37* and *TaPRR73* revealed differences in the promoter regions that could be the underlying reason for differences in expression. Zawaski et al. (2012) reported two putative PHOTOPERIOD RESPONSE 1 (PHOR1) orthologs, *PtPHOR1_1 and PtPHOR1_2*, in *Populus. PtPHOR1_1* was most highly expressed in, and restricted to, the roots, whereas *PtPHOR1_2* was more uniformly expressed throughout all plant tissues with similar effects in aerial and below-ground tissues. We therefore speculate that *TaPRR37* mainly affects aerial tissues and that *TaPRR73* has effects on both aerial parts and roots. Many important agronomic traits, such as drought and salinity tolerances, are related to root development, therefore justifying further investigation of the functions of *TaPRR73* in roots. Functional divergence of paralogs might result from differences in the promotor regions. The functions of *TaPRR37* and *TaPRR73* are either similar or diverged in functional complementarity, but work together in plant growth and development.

*PRR37* has been clearly recognized and widely employed in wheat and rice breeding and has made enormous contributions worldwide. Here, we identified functions of *PRR73* that could be applied in crop improvement. For example, favorable haplotypes of *TaPRR73-A1* were selected in past breeding programs. Although, no favorable allele was found in the D genome of hexapolid wheat, perhaps due to the domestication bottleneck associated with hexaploidisation, more diversity may be present the diploid progenitor *Ae. tauschii*, which is known to be rich in genetic diversity (Jia et al., 2013). Any favorable allele in the species can easily be transferred to common wheat by development of synthetic wheat followed by introgression to agronomically adapted cultivars.

### Development of an efficient platform to mine paralogous gene function

Plant genome sequencing has revealed that many plant genomes have paralogous gene sets. However, their individual functions are rarely reported (Xu et al., 2015). In the present study, we investigated *TaPRR73*, a paralog of the well-known *Ppd1* gene series, as a target gene, and employed a series of approaches (including transformation experiments, expression analysis, haplotype analysis, and association analysis) to mine its function. We transferred about 4000 wheat transcription factors to rice to observe their functions by over-expression. We constructed a core collection and a series of introgression lines for association analysis. With rapid advances in plant genomics techniques, increasing numbers of platforms and databases are available for paralogous gene analysis. RNA-seq databases (Wang et al., 2009; Bansal et al., 2014) also provide a platform to detect paralogous gene expression patterns. Techniques for achieving high transformation rates in rice (Duan et al., 2012), wheat (Ishida et al., 2015), and other crops provide effective platforms to confirm gene function. Genotypes of core collections and ILs generated from re-sequencing and high density SNP arrays together with their phenotypes provide a platform for GWAS (Topol and Frazer, 2007). All of these platforms and methods will accelerate mining of paralogous gene function.

## Author contributions

WZ, JJ, and BW designed the study. WZ, GZ, LG, XK, and ZG collected data and performed the research. WZ, JJ Wrote the paper.

### Conflict of interest statement

The authors declare that the research was conducted in the absence of any commercial or financial relationships that could be construed as a potential conflict of interest.

## Abbreviations

*PRR*: pseudo-response regulator
PR: pseudoreceiver
IR: intermediate region
CCT, CONSTANS, CO-like: TOC1
RIL: recombinant inbred line
ILs: introgression lines
LDs: long day conditions
SDs: short day conditions
PVE: phenotypic variation explained.
