# Supplementary material for: Functional Studies of Heading Date-Related Gene TaPRR73, a Paralog of Ppd1 in Common Wheat
Source: Front Plant Sci. 2016 Jun 1;7:772. doi: 10.3389/fpls.2016.00772 (PMC4887500; doi:10.3389/fpls.2016.00772)
Supplement: Supplementary file 1 [file DataSheet1.docx]

**Functional study of heading date related gene *TaPRR73*, a paralog of *Ppd1* in wheat**

| Supplementary Table 1-1. Distribution of *TaPRR73-A1* haplotypes in wild species | | | | | |
| --- | --- | --- | --- | --- | --- |
| Number | Genome type | Haplotype | Number | Genome type | Haplotype |
| 16436① | AABB | HapI | 17502 | AABB | HapⅡ |
| 16452② | AABB | HapI | 17503 | AABB | HapⅡ |
| 16459② | AABB | HapI | 17504 | AABB | HapⅡ |
| 16460① | AABB | HapI | 17505 | AABB | HapⅡ |
| 16435① | AABB | HapⅡ | 17506 | AABB | HapⅡ |
| 16439① | AABB | HapⅡ | 17507 | AABB | HapⅡ |
| 16440① | AABB | HapⅡ | 17508 | AABB | HapⅡ |
| 16440② | AABB | HapⅡ | 17509 | AABB | HapⅡ |
| 16445① | AABB | HapⅡ | 17514 | AABB | HapⅡ |
| 16447① | AABB | HapⅡ | 17515 | AABB | HapⅡ |
| 16447② | AABB | HapⅡ | 17516 | AABB | HapⅡ |
| 16451① | AABB | HapⅡ | 17517 | AABB | HapⅡ |
| 16452① | AABB | HapⅡ | 17518 | AABB | HapⅡ |
| 16456① | AABB | HapⅡ | 17519 | AABB | HapⅡ |
| 16458② | AABB | HapⅡ | 17520① | AABB | HapⅡ |
| 16459① | AABB | HapⅡ | 17510 | AABB | HapⅡ |
| 16461② | AABB | HapⅡ | 17511 | AABB | HapⅡ |
| 16462② | AABB | HapⅡ | 17512 | AABB | HapⅡ |
| 16464① | AABB | HapⅡ | 17513 | AABB | HapⅡ |
| 16466① | AABB | HapⅡ | A-1 | AA | HapIII |
| 16466② | AABB | HapⅡ | A-2 | AA | HapIII |
| 16467① | AABB | HapⅡ | A-3 | AA | HapIII |
| 16467② | AABB | HapⅡ | A-4 | AA | HapIII |
| 17497 | AABB | HapⅡ | A-5 | AA | HapIII |
| 17498 | AABB | HapⅡ | A-6 | AA | HapⅣ |
| 17499 | AABB | HapⅡ | A-7 | AA | HapⅣ |
| 17500 | AABB | HapⅡ | A-8 | AA | HapⅣ |
| 17501 | AABB | HapⅡ | A-9 | AA | HapⅣ |

| Supplementary Table 2-1. Distribution of *TaPRR73-B1* haplotypes in wild species | | | | | |
| --- | --- | --- | --- | --- | --- |
| Number | Genome type | Haplotype | Number | Genome type | Haplotype |
| 16459① | AABB | HapI | 17503 | AABB | HapI |
| 16452② | AABB | HapI | 17504 | AABB | HapI |
| 16466① | AABB | HapI | 17506 | AABB | HapI |
| 16467① | AABB | HapI | 17507 | AABB | HapI |
| 16456① | AABB | HapI | 17511 | AABB | HapI |
| 16451① | AABB | HapI | 17512 | AABB | HapI |
| 16452① | AABB | HapI | 17513 | AABB | HapI |
| 16461② | AABB | HapI | 17521① | AABB | HapI |
| 16477① | AABB | HapI | 17521② | AABB | HapI |
| 16477② | AABB | HapI | 17522① | AABB | HapI |
| 16436① | AABB | HapI | 17523② | AABB | HapI |
| 16466② | AABB | HapI | 17524② | AABB | HapI |
| 16447② | AABB | HapI | 17525① | AABB | HapI |
| 16440① | AABB | HapI | 17525② | AABB | HapI |
| 16457① | AABB | HapI | 17526① | AABB | HapI |
| 16457② | AABB | HapI | 17526② | AABB | HapI |
| 16466② | AABB | HapI | 17527① | AABB | HapI |
| 17514 | AABB | HapI | 17528① | AABB | HapI |
| 17515 | AABB | HapI | 17528② | AABB | HapI |
| 17516 | AABB | HapI | 17529① | AABB | HapI |
| 17517 | AABB | HapI | 17529② | AABB | HapI |
| 17518 | AABB | HapI | 17530① | AABB | HapI |
| 17519 | AABB | HapI | 17530② | AABB | HapI |
| 17520① | AABB | HapI | 17531① | AABB | HapI |
| 17520② | AABB | HapI | 17532① | AABB | HapI |
| 17431① | AABB | HapI | 17533① | AABB | HapI |
| 17432② | AABB | HapI | 17533② | AABB | HapI |
| 17434① | AABB | HapI | 17534① | AABB | HapI |
| 17434② | AABB | HapI | 17534② | AABB | HapI |
| 17435① | AABB | HapI | 17535① | AABB | HapI |
| 17435② | AABB | HapI | 17535② | AABB | HapI |
| 17436① | AABB | HapI | 17536① | AABB | HapI |
| 17436② | AABB | HapI | 17536② | AABB | HapI |
| 17437① | AABB | HapI | 17537① | AABB | HapI |
| 17437② | AABB | HapI | 17537② | AABB | HapI |
| 17438① | AABB | HapI | 17471 | AABB | HapI |
| 17439② | AABB | HapI | 17472 | AABB | HapI |
| 17442② | AABB | HapI | 17473 | AABB | HapI |
| 17443① | AABB | HapI | 17474 | AABB | HapI |

| Supplementary Table 2-2. Distribution of *TaPRR73-B1* haplotypes in wild species | | | | | |
| --- | --- | --- | --- | --- | --- |
| Number | Genome type | Haplotype | Number | Genome type | Haplotype |
| 17443② | AABB | HapI | 17476 | AABB | HapI |
| 17444① | AABB | HapI | 17477 | AABB | HapI |
| 17444② | AABB | HapI | 17478 | AABB | HapI |
| 17445② | AABB | HapI | 17479 | AABB | HapI |
| 17446① | AABB | HapI | 17480 | AABB | HapI |
| 17446② | AABB | HapI | 17481 | AABB | HapI |
| 17447② | AABB | HapI | 17484 | AABB | HapI |
| 17449① | AABB | HapI | 17486 | AABB | HapI |
| 17449② | AABB | HapI | 17487 | AABB | HapI |
| 17450① | AABB | HapI | 17488 | AABB | HapI |
| 17450② | AABB | HapI | 17489 | AABB | HapI |
| 17451① | AABB | HapI | 17491 | AABB | HapI |
| 17452② | AABB | HapI | 17492 | AABB | HapI |
| 17453② | AABB | HapI | 17493 | AABB | HapI |
| 17454① | AABB | HapI | 17494 | AABB | HapI |
| 17454② | AABB | HapI | 17495 | AABB | HapI |
| 17455 | AABB | HapI | 17496 | AABB | HapI |
| 17456 | AABB | HapI | 17497 | AABB | HapI |
| 17457 | AABB | HapI | 17498 | AABB | HapI |
| 17459 | AABB | HapI | 17499 | AABB | HapⅡ |
| 17460 | AABB | HapI | 17500 | AABB | HapI |
| 17461 | AABB | HapI | 17501 | AABB | HapI |
| 17462 | AABB | HapI | 17502 | AABB | HapI |
| 17464 | AABB | HapI | 17505 | AABB | HapⅡ |
| 17465 | AABB | HapI | 16460① | AABB | HapⅡ |
| 17466 | AABB | HapI | 17431② | AABB | HapⅡ |
| 17467 | AABB | HapI | 17433② | AABB | HapⅡ |
| 17468 | AABB | HapI | 17441② | AABB | HapⅡ |
| 17469 | AABB | HapI | 17442① | AABB | HapⅡ |
| 17470 | AABB | HapI |  |  |  |

| Supplementary Table 3. Haplotypes of *TaPRR73-A1* and *TaPRR73-B1* in landraces (L) and modern cultivars (M) from ten major wheat plant regions | | | | | |
| --- | --- | --- | --- | --- | --- |
| Accessions | L or M | Regions | *TaPRR73-A1* | *TaPRR73-B1* | |
| Dingxingzhai | L | NW | HapⅡ | | HapⅠ |
| Baixiaomai | L | NW | HapⅡ | | HapⅡ |
| Chadianhong | L | NW | HapⅡ | | HapⅠ |
| Gezhoumai | L | NW | HapⅡ | | HapⅠ |
| Shanhongmai | L | NW | HapⅡ | | HapⅠ |
| Changfeng 4 | M | NW | HapⅠ | | HapⅠ |
| Changfeng 6 | M | NW | HapⅠ | | HapⅠ |
| Zaosui 30 | M | NW | HapⅠ | | HapⅠ |
| Jingshuang 16 | M | NW | HapⅠ | | HapⅠ |
| Jinghua 2 | M | NW | HapⅠ | | HapⅠ |
| Yuanxie 62 | M | NW | HapⅡ | | HapⅠ |
| Zhongyou 9507 | M | NW | HapⅠ | | HapⅠ |
| Hanxuan 10 | M | NW | HapⅡ | | HapⅠ |
| Pingyang 181 | M | NW | HapⅡ | | HapⅠ |
| Jinmai 47 | M | NW | HapⅠ | | HapⅡ |
| Jing 411 | M | NW | HapⅠ | | HapⅠ |
| Beijing 837 | M | NW | HapⅠ | | HapⅠ |
| Huabei 187 | M | NW | HapⅠ | | HapⅠ |
| Hanxuan 3 | M | NW | HapⅠ | | HapⅠ |
| Nongda 311 | M | NW | HapⅠ | | HapⅠ |
| Yanda 1817 | M | NW | HapⅡ | | HapⅠ |
| Beijing 8 | M | NW | HapⅡ | | HapⅠ |
| Keyi 26 | M | NW | HapⅡ | | HapⅠ |
| Pindong 34 | M | NW | HapⅠ | | HapⅡ |
| Yuandong 3 | M | NW | HapⅠ | | HapⅠ |
| Jingdong 8 | M | NW | HapⅠ | | HapⅠ |
| Beijing 0045 | M | NW | HapⅠ | | HapⅡ |
| Jingken 49 | M | NW | HapⅡ | | HapⅠ |
| Jingdong 22 | M | NW | HapⅠ | | HapⅠ |
| Nongda 211 | M | NW | HapⅡ | | HapⅠ |
| Zhongmai 175 | M | NW | HapⅠ | | HapⅠ |
| Zhongmai 415 | M | NW | HapⅠ | | HapⅠ |
| Zhongyun 1 | M | NW | HapⅠ | | HapⅠ |
| Zhongmai 13 | M | NW | HapⅡ | | HapⅠ |
| Fengkang 8 | M | NW | HapⅠ | | HapⅠ |
| Dingxingzhai | L | NS | HapⅡ | | HapⅠ |
| Dahongmai | L | NS | HapⅠ | | HapⅡ |
| Xiaobaimang | L | NS | HapⅠ | | HapⅠ |
| Xiaobaimang | L | NS | HapⅠ | | - |
| Jinghong5 | M | NS | HapⅠ | | HapⅠ |
| Sankecun | L | MLYW | HapⅠ | | HapⅡ |
| Lanxizaoxiaomai | L | MLYW | HapⅡ | | HapⅡ |
| Heshangmai | L | MLYW | HapⅡ | | HapⅠ |
| Jiyumai | L | MLYW | HapⅡ | | HapⅠ |
| Hongheshangtou | L | MLYW | HapⅡ | | HapⅠ |
| Jiangdongmen | L | MLYW | HapⅡ | | HapⅠ |
| Huxumai | L | MLYW | HapⅡ | | HapⅠ |
| Liuzhutou | L | MLYW | HapⅡ | | HapⅠ |
| Nuomai | L | MLYW | HapⅡ | | HapⅠ |
| Xiaoziganzi | L | MLYW | HapⅠ | | - |
| Heputou | L | MLYW | HapⅡ | | HapⅠ |
| Sifangmai | L | MLYW | HapⅡ | | HapⅠ |
| Baicimai | L | MLYW | HapⅡ | | HapⅠ |
| Hongkeyoumang | L | MLYW | HapⅠ | | HapⅠ |
| Honghuazao | L | MLYW | HapⅡ | | HapⅠ |
| Dabaimai | L | MLYW | HapⅡ | | HapⅠ |
| Heshangtou | L | MLYW | HapⅡ | | HapⅠ |
| Huoshaotian | L | MLYW | HapⅡ | | HapⅠ |
| Huajiechangbaican | L | MLYW | HapⅡ | | HapⅠ |
| Meiqianwu | L | MLYW | HapⅡ | | HapⅡ |
| Nanda 2419 | M | MLYW | HapⅡ | | HapⅠ |
| Yangmai 5 | M | MLYW | HapⅠ | | HapⅡ |
| Yangmai 12 | M | MLYW | HapⅠ | | HapⅡ |
| Sumai 3 | M | MLYW | HapⅠ | | HapⅡ |
| Nanda 96co76 | M | MLYW | HapⅠ | | HapⅡ |
| Xinyang 12 | M | MLYW | HapⅡ | | HapⅡ |
| Anhui 11 | M | MLYW | HapⅡ | | HapⅠ |
| Exi 84-1031 | M | MLYW | HapⅠ | | HapⅠ |
| Paozimai | M | MLYW | HapⅡ | | HapⅠ |
| Emai 11 | M | MLYW | HapⅠ | | HapⅠ |
| Wanmai 31 | M | MLYW | HapⅠ | | HapⅠ |
| Wanmai 33 | M | MLYW | HapⅠ | | HapⅠ |
| Ningmai 9 | M | MLYW | HapⅡ | | HapⅠ |
| Sumai 6 | M | MLYW | HapⅠ | | HapⅠ |
| Emai 12 | M | MLYW | HapⅡ | | HapⅠ |
| Zhenmai 3 | M | MLYW | HapⅠ | | HapⅡ |
| Emai 15 | M | MLYW | HapⅡ | | HapⅡ |
| Shengyuan 3 | M | MLYW | HapⅠ | | HapⅡ |
| Emai 16 | M | MLYW | HapⅠ | | HapⅡ |
| Emai 17 | M | MLYW | HapⅠ | | HapⅠ |
| Emai 18 | M | MLYW | HapⅠ | | HapⅠ |
| Emai 19 | M | MLYW | HapⅡ | | HapⅡ |
| Yangmai 13 | M | MLYW | HapⅠ | | HapⅡ |
| Zhenmai 4 | M | MLYW | HapⅡ | | HapⅠ |
| Yangmai 14 | M | MLYW | HapⅡ | | HapⅠ |
| Yangmai 15 | M | MLYW | HapⅠ | | HapⅡ |
| Yangmai 16 | M | MLYW | HapⅠ | | HapⅡ |
| Zhenmai 5 | M | MLYW | HapⅡ | | HapⅡ |
| Ningmai 12 | M | MLYW | HapⅠ | | HapⅠ |
| Zhenmai 17 | M | MLYW | HapⅡ | | HapⅡ |
| Zhenmai 6 | M | MLYW | HapⅡ | | HapⅠ |
| ningmai 14 | M | MLYW | HapⅡ | | HapⅡ |
| Zhenmai 168 | M | MLYW | HapⅠ | | HapⅡ |
| Yangmai 18 | M | MLYW | HapⅡ | | HapⅠ |
| Ningnuomai 1 | M | MLYW | HapⅠ | | HapⅡ |
| Youmangbaifu | L | NeS | HapⅡ | | HapⅠ |
| Huoqiu | L | NeS | HapⅡ | | HapⅠ |
| Kefeng 3 | M | NeS | HapⅠ | | - |
| Jichun 1026 | M | NeS | HapⅠ | | HapⅡ |
| Hezuo 2 | M | NeS | HapⅠ | | HapⅠ |
| Songhuajiang 1 | M | NeS | HapⅠ | | HapⅠ |
| Heifu 84S1378 | M | NeS | HapⅠ | | HapⅠ |
| Xinkehan 9 | M | NeS | HapⅠ | | HapⅠ |
| Longfu 91B-569 | M | NeS | HapⅡ | | HapⅠ |
| Kenhong 14 | M | NeS | HapⅠ | | HapⅠ |
| Qigongmai | L | SCW | HapⅡ | | HapⅠ |
| Tumai | L | SCW | HapⅡ | | HapⅠ |
| Shanglinxiaomai | L | SCW | HapⅡ | | HapⅠ |
| Bendixiaomai | L | SCW | HapⅡ | | HapⅠ |
| Kangmai | L | SCW | HapⅡ | | HapⅠ |
| Ronganxiaomai | L | SCW | HapⅡ | | HapⅠ |
| Damoxu | L | SCW | HapⅡ | | HapⅠ |
| Wumangmai | L | SCW | HapⅡ | | HapⅡ |
| Jinmai2148 | M | SCW | HapⅠ | | HapⅡ |
| Taizhong23 | M | SCW | HapⅠ | | HapⅠ |
| Baituzimai | L | YHW | HapⅡ | | HapⅠ |
| Zijihong | L | YHW | HapⅠ | | HapⅠ |
| Youzimai | L | YHW | HapⅡ | | HapⅠ |
| Hongmangyouzimai | L | YHW | HapⅡ | | HapⅠ |
| Sanyuehong | L | YHW | HapⅡ | | HapⅠ |
| Tumangmai | L | YHW | HapⅡ | | HapⅠ |
| Mazhaimai | L | YHW | HapⅡ | | HapⅠ |
| Xiaofushou | L | YHW | HapⅡ | | HapⅠ |
| Chiyazao | L | YHW | HapⅡ | | HapⅠ |
| Youzitou | L | YHW | HapⅡ | | HapⅠ |
| Hongqiangchang | L | YHW | HapⅡ | | HapⅠ |
| banjiemang | L | YHW | HapⅡ | | HapⅠ |
| Dazibai | L | YHW | HapⅠ | | HapⅠ |
| Youmangsaogudan | L | YHW | HapⅡ | | HapⅠ |
| Dakoumai | L | YHW | HapⅡ | | HapⅠ |
| Louguding | L | YHW | HapⅡ | | HapⅠ |
| Huangguaxian | L | YHW | HapⅡ | | HapⅠ |
| Fumai | L | YHW | HapⅡ | | HapⅠ |
| Hongquanmang | L | YHW | HapⅡ | | HapⅠ |
| Baiyoumai | L | YHW | HapⅡ | | HapⅠ |
| Baipu | L | YHW | HapⅡ | | HapⅠ |
| Laomai | L | YHW | HapⅡ | | HapⅡ |
| Shuangji 4 | M | YHW | HapⅡ | | HapⅡ |
| Lumai 14 | M | YHW | HapⅠ | | HapⅠ |
| Yumai 18 | M | YHW | HapⅠ | | HapⅡ |
| Changnong 339-5-1 | M | YHW | HapⅠ | | HapⅠ |
| Changwei 18 | M | YHW | HapⅡ | | HapⅠ |
| Han 99-6143 | M | YHW | HapⅡ | | HapⅡ |
| Neixiang 188 | M | YHW | HapⅠ | | HapⅡ |
| Xiaoyan 54 | M | YHW | HapⅠ | | HapⅠ |
| Gaoyou 503 | M | YHW | HapⅠ | | HapⅠ |
| Jinmai 54 | M | YHW | HapⅠ | | - |
| Bainong 3217 | M | YHW | HapⅠ | | HapⅠ |
| Xuzhou 14 | M | YHW | HapⅡ | | HapⅠ |
| Shannong 1870 | M | YHW | HapⅠ | | HapⅠ |
| Wenmai 6 | M | YHW | HapⅠ | | HapⅠ |
| Weimai 4 | M | YHW | HapⅡ | | HapⅠ |
| Bima 4 | M | YHW | HapⅡ | | HapⅠ |
| Jinan 13 | M | YHW | HapⅠ | | HapⅠ |
| Jingyang 60 | M | YHW | HapⅡ | | HapⅠ |
| Xinong 6028 | M | YHW | HapⅠ | | HapⅠ |
| Yumai 8 | M | YHW | HapⅡ | | HapⅠ |
| Shanhan 8675 | M | YHW | HapⅠ | | HapⅠ |
| Heibao 4 | M | YHW | HapⅠ | | HapⅠ |
| Teng S15 | M | YHW | HapⅡ | | HapⅠ |
| Lankao 90(6)52-24 | M | YHW | HapⅠ | | HapⅡ |
| Shan 354 | M | YHW | HapⅠ | | HapⅠ |
| Jimai 6 | M | YHW | HapⅠ | | HapⅠ |
| Songcheng 9411 | M | YHW | HapⅠ | | HapⅠ |
| Fuzhuang 30 | M | YHW | HapⅡ | | HapⅠ |
| Xinxiang 9178 | M | YHW | HapⅡ | | HapⅠ |
| Zhengmai 9201 | M | YHW | HapⅠ | | HapⅡ |
| Linkang5027 | M | YHW | HapⅠ | | HapⅡ |
| Shi 4185 | M | YHW | HapⅡ | | HapⅠ |
| Xingmai 1 | M | YHW | HapⅠ | | HapⅠ |
| Yan 893 | M | YHW | HapⅠ | | HapⅡ |
| Kanghuixianhong | L | YHW | HapⅠ | | HapⅠ |
| Youbao | L | YHW | HapⅠ | | HapⅠ |
| Aifeng 3 | M | YHW | HapⅡ | | HapⅡ |
| Anyang 1 | M | YHW | HapⅡ | | HapⅠ |
| Yannong 15 | M | YHW | HapⅠ | | HapⅠ |
| Xian 83（104）-11“s" | M | YHW | HapⅡ | | HapⅠ |
| Yuanzhu | L | YHW | HapⅠ | | HapⅡ |
| Shangluo 81(2)4-19-23 | M | YHW | HapⅠ | | HapⅡ |
| Wanyuan 66 | M | YHW | HapⅠ | | HapⅠ |
| Wan 7107 | M | YHW | HapⅠ | | HapⅠ |
| Wenmai 4 | M | YHW | HapⅠ | | HapⅠ |
| Yumai 49 | M | YHW | HapⅠ | | HapⅠ |
| Xinmai 9 | M | YHW | HapⅠ | | HapⅠ |
| Yanzhan 4110 | M | YHW | HapⅠ | | HapⅡ |
| Yanzhan 1 | M | YHW | HapⅠ | | HapⅡ |
| Zhengmai 9023 | M | YHW | HapⅠ | | HapⅠ |
| Zhoumai 16 | M | YHW | HapⅠ | | HapⅠ |
| Yumai 70-36 | M | YHW | HapⅠ | | HapⅡ |
| Yunong 949 | M | YHW | HapⅡ | | HapⅠ |
| Aikang 58 | M | YHW | HapⅠ | | HapⅠ |
| Zhou 18 | M | YHW | HapⅠ | | HapⅠ |
| Zhoumai 22 | M | YHW | HapⅠ | | HapⅠ |
| Luomai 23 | M | YHW | HapⅡ | | HapⅠ |
| Dong 2-23 | M | YHW | HapⅡ | | HapⅡ |
| Dong 1-23 | M | YHW | HapⅠ | | HapⅠ |
| Huaichuan 916 | M | YHW | HapⅠ | | HapⅠ |
| FS 056 | M | YHW | HapⅠ | | HapⅠ |
| Jiaomai 266 | M | YHW | HapⅠ | | HapⅠ |
| FS 059 | M | YHW | HapⅠ | | HapⅠ |
| Dong 1-32 | M | YHW | HapⅠ | | HapⅠ |
| Zhongren 2 | M | YHW | HapⅠ | | HapⅠ |
| Luomai 26 | M | YHW | HapⅠ | | HapⅡ |
| Zhongjiao 1 | M | YHW | HapⅡ | | HapⅡ |
| Zhongjiao 2 | M | YHW | HapⅠ | | HapⅡ |
| Zhongjiao 3 | M | YHW | HapⅠ | | HapⅡ |
| Zhongluo 08-3 | M | YHW | HapⅡ | | HapⅡ |
| Zhongluo 08-2 | M | YHW | HapⅡ | | HapⅠ |
| Zhongluo 08-1 | M | YHW | HapⅠ | | HapⅠ |
| Kenong 199 | M | YHW | HapⅡ | | HapⅡ |
| FS 198 | M | YHW | HapⅠ | | HapⅠ |
| Dong 3-6 | M | YHW | HapⅡ | | HapⅡ |
| Dong 2-8 | M | YHW | HapⅡ | | HapⅡ |
| Jining 13 | M | YHW | HapⅠ | | HapⅠ |
| Zimai 12 | M | YHW | HapⅡ | | HapⅠ |
| Weimai 6 | M | YHW | HapⅠ | | HapⅠ |
| Yannong 22 | M | YHW | HapⅠ | | HapⅠ |
| Taishan 9818 | M | YHW | HapⅠ | | HapⅡ |
| Jimai 21 | M | YHW | HapⅠ | | HapⅠ |
| Weimai 8 | M | YHW | HapⅠ | | HapⅠ |
| Taishan 21 | M | YHW | HapⅡ | | HapⅠ |
| Liaomai 16 | M | YHW | HapⅡ | | HapⅠ |
| Jimai 19 | M | YHW | HapⅠ | | HapⅠ |
| Yannong 24 | M | YHW | HapⅠ | | HapⅠ |
| Taishan 23 | M | YHW | HapⅡ | | HapⅠ |
| Jining 16 | M | YHW | HapⅠ | | HapⅠ |
| Linmai 2 | M | YHW | HapⅠ | | HapⅠ |
| Liangxing 99 | M | YHW | HapⅠ | | HapⅡ |
| Yannong 23 | M | YHW | HapⅠ | | HapⅠ |
| Jimai 20 | M | YHW | HapⅠ | | HapⅠ |
| Shannong 12 | M | YHW | HapⅠ | | HapⅠ |
| Shannong 15 | M | YHW | HapⅠ | | HapⅡ |
| Wennong 6 | M | YHW | HapⅠ | | HapⅠ |
| Linmai 4 | M | YHW | HapⅠ | | HapⅠ |
| Taimai 1 | M | YHW | HapⅡ | | HapⅡ |
| Jimai 22 | M | YHW | HapⅠ | | HapⅡ |
| Luyuan 301 | M | YHW | HapⅠ | | HapⅠ |
| Zhouyuan9369 | M | YHW | HapⅠ | | HapⅠ |
| Tainong 18 | M | YHW | HapⅠ | | HapⅠ |
| Liangxing 66 | M | YHW | HapⅠ | | HapⅡ |
| Kexin 9 | M | YHW | HapⅠ | | HapⅠ |
| Jimai36 | M | YHW | HapⅠ | | HapⅠ |
| Shi 5093 | M | YHW | HapⅠ | | HapⅠ |
| Shijiazhuang 8 | M | YHW | HapⅠ | | HapⅠ |
| Han 6172 | M | YHW | HapⅠ | | HapⅠ |
| Shimai 12 | M | YHW | HapⅠ | | HapⅠ |
| Shiluan 02-1 | M | YHW | HapⅡ | | HapⅠ |
| Shimai 15 | M | YHW | HapⅠ | | HapⅠ |
| Shiyou 17 | M | YHW | HapⅠ | | HapⅠ |
| Ji 5265 | M | YHW | HapⅠ | | HapⅠ |
| Shimai 18 | M | YHW | HapⅡ | | HapⅠ |
| Gaoyou 2018 | M | YHW | HapⅠ | | HapⅠ |
| Shimai 19 | M | YHW | HapⅡ | | HapⅠ |
| Shiyou 20 | M | YHW | HapⅠ | | HapⅠ |
| Shanyou 225 | M | YHW | HapⅠ | | HapⅠ |
| Shan 160 | M | YHW | HapⅠ | | HapⅠ |
| Xiaoyan 22 | M | YHW | HapⅡ | | HapⅠ |
| Shanmai 150 | M | YHW | HapⅠ | | - |
| Shan 253 | M | YHW | HapⅠ | | HapⅠ |
| Xinong 979 | M | YHW | HapⅠ | | HapⅠ |
| Shanmai 159 | M | YHW | HapⅡ | | HapⅠ |
| Huaimai 22 | M | YHW | HapⅠ | | HapⅠ |
| Zhongyou 206 | M | YHW | HapⅠ | | HapⅠ |
| Bima 1 | M | YHW | HapⅡ | | HapⅠ |
| Chayazheda 29 | M | QTSW | HapⅡ | | HapⅡ |
| Changgongfangxingmai | L | QTSW | HapⅡ | | HapⅠ |
| Rikaze 54 | M | QTSW | HapⅡ | | HapⅡ |
| Gaoyuan 602 | M | QTSW | HapⅠ | | HapⅡ |
| Gaoyuan 338 | M | QTSW | HapⅡ | | - |
| Lanhuamai | L | NwS | HapⅡ | | HapⅠ |
| Lianglaiyoubaipixiaomai | L | NwS | HapⅡ | | HapⅡ |
| Shanmai | L | NwS | HapⅡ | | HapⅡ |
| Biyumai | L | NwS | HapⅠ | | HapⅡ |
| Ganmai 8 | M | NwS | HapⅡ | | HapⅠ |
| Qingfeng 1 | M | NwS | HapⅡ | | HapⅠ |
| Gao 38 | M | NwS | HapⅠ | | HapⅠ |
| Ningchun 13 | M | NwS | HapⅠ | | HapⅠ |
| Ningchun 10 | M | NwS | HapⅡ | | HapⅡ |
| Ningchun 4 | M | NwS | HapⅠ | | HapⅠ |
| Yunnanxiaomai | L | SwW | HapⅡ | | HapⅠ |
| Jiangmai | L | SwW | HapⅡ | | HapⅠ |
| Huanxiangguo | L | SwW | HapⅡ | | HapⅠ |
| Changmangyingkemai | L | SwW | HapⅡ | | HapⅠ |
| Zhongguochun | L | SwW | HapⅡ | | HapⅠ |
| Wuxumai | L | SwW | HapⅡ | | HapⅠ |
| Baihuamai | L | SwW | HapⅡ | | - |
| Yuqiumai | L | SwW | HapⅡ | | HapⅠ |
| Maikou | L | SwW | HapⅡ | | - |
| Caijiangmai | L | SwW | HapⅡ | | - |
| Hongpi | L | SwW | HapⅡ | | HapⅠ |
| Zipi | L | SwW | HapⅡ | | - |
| Baimaizi | L | SwW | HapⅡ | | HapⅠ |
| Bendimai | L | SwW | HapⅡ | | HapⅠ |
| Hongkejiang | L | SwW | HapⅡ | | HapⅠ |
| Kangdingxiaomai | L | SwW | HapⅡ | | HapⅠ |
| Sanyuanmai | L | SwW | HapⅡ | | HapⅡ |
| Tuotuomai | L | SwW | HapⅡ | | HapⅡ |
| Yangmai | L | SwW | HapⅡ | | HapⅡ |
| Neijiang 31 | M | SwW | HapⅡ | | - |
| Zhen 662-525-2 | M | SwW | HapⅠ | | HapⅡ |
| Jinshajiang 1 | M | SwW | HapⅡ | | HapⅠ |
| Xichang 76-9 | M | SwW | HapⅠ | | HapⅠ |
| Mianyang 86-11 | M | SwW | HapⅡ | | HapⅠ |
| Chuangnong 12 | M | SwW | HapⅡ | | HapⅠ |
| Mianyang 79-2 | M | SwW | HapⅡ | | HapⅠ |
| Chuan 84-7045 | M | SwW | HapⅠ | | HapⅠ |
| Fan 6 | M | SwW | HapⅡ | | HapⅠ |
| Chuanmai 8 | M | SwW | HapⅠ | | HapⅠ |
| Xingyi 4 | M | SwW | HapⅠ | | HapⅠ |
| Mianyang 15 | M | SwW | HapⅡ | | HapⅠ |
| Mianyang 19 | M | SwW | HapⅡ | | HapⅠ |
| Mianyang 20 | M | SwW | HapⅡ | | HapⅠ |
| Kashebaipi | L | SkWS | HapⅠ | | HapⅠ |
| Yutiandaomaizi | L | SkWS | HapⅠ | | HapⅠ |
| Hongdongmai | L | SkWS | HapⅡ | | HapⅠ |
| Hongchunmai | L | SkWS | HapⅡ | | HapⅠ |
| Xinjiang 1 | L | SkWS | HapⅠ | | - |
| Xinchun 6 | M | SkWS | HapⅡ | | HapⅠ |
| Xinchun 8 | M | SkWS | HapⅡ | | HapⅠ |
| Xinchun 11 | M | SkWS | HapⅡ | | HapⅠ |

Ten major wheat plant regions of China. NW: Northern winter wheat region; NS: Northern spring wheat region; MLYW: Middle and low yangtze valley winter wheat region; NeS: Northeastern spring wheat region; SCW: South china winter wheat region; YHW: Yellow and huai river winter wheat region; QTSW: Qinghai-Tibet spring-winter wheat region; NwS: Northwestern spring wheat region; SwW: Southwestern winter wheat region; SkWS: Sinkiang winter-spring wheat region.


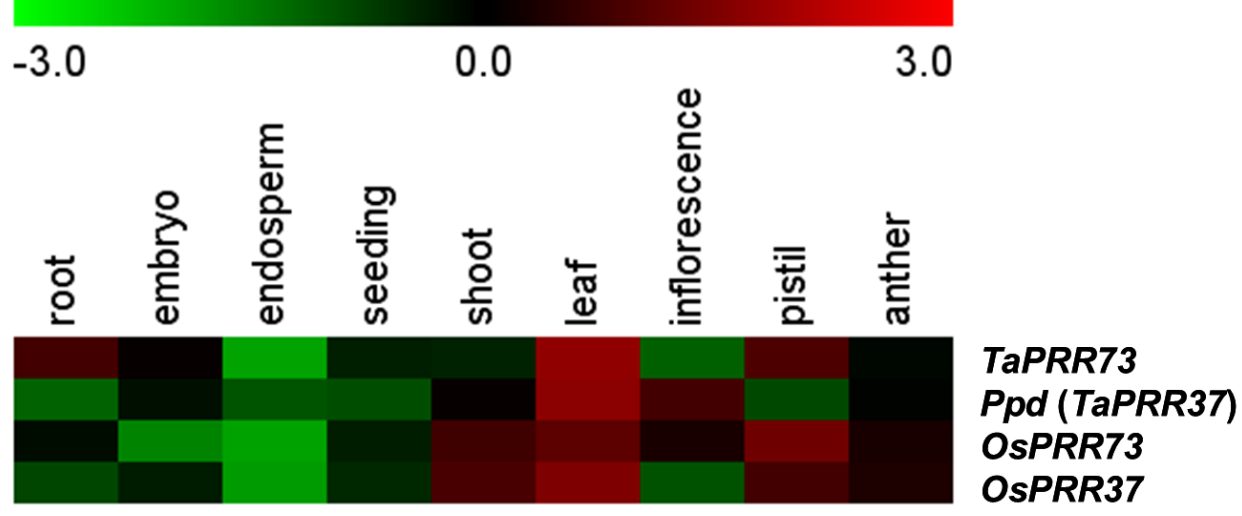


Supplementary Figure 1**.** Comparative *TaPRR73* expression based on *in silico* data for *Ppd, OsPRR73* and *OsPRR37* in different organs. The microarray data were obtained from Genevestigator (Zimmermann et al., 2008) and standardized by Z scores (Schmid et al.*,* 2005). Green color represents the lowest expression level and red color the highest. All four genes were most highly expressed in leaves, and least expressed in endosperm. Expression levels of *TaPRR73* in roots were higher than *OsPRR37*, *OsPRR73* and *TaPRR37.*


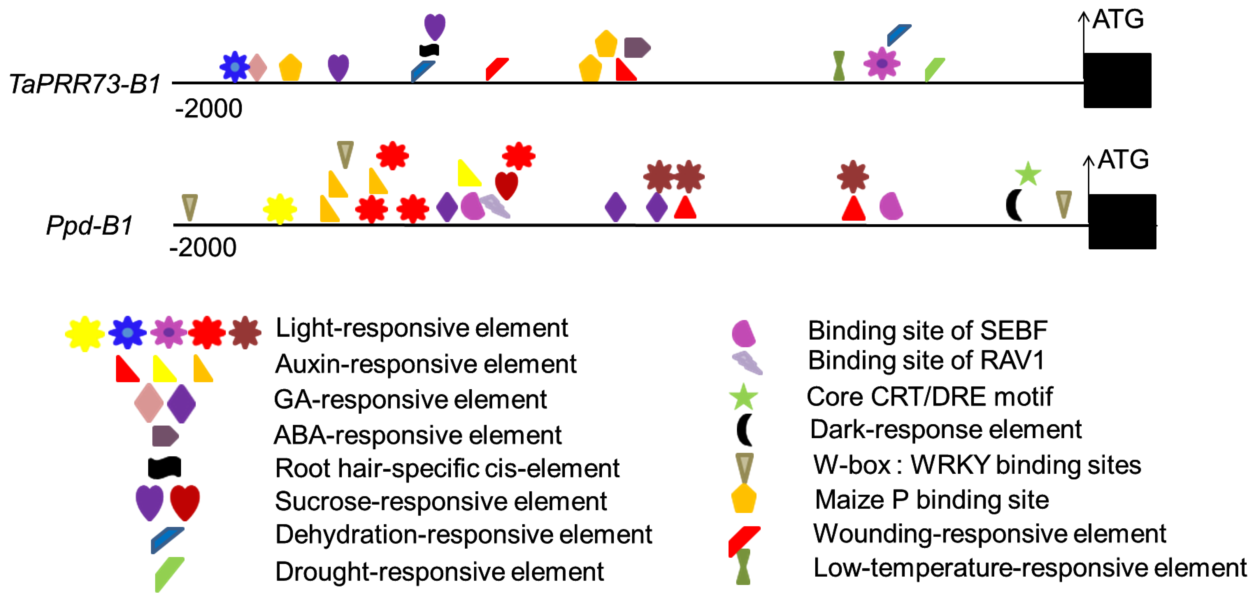


Supplementary Figure **2.** Cis-regulatory elements in the promoter regions of *TaPRR73-B1* and *Ppd-B1*.


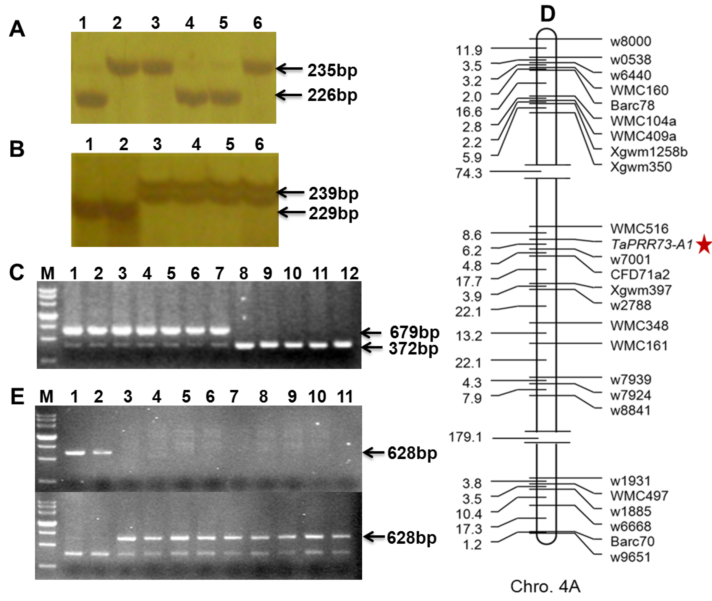


Supplementary Figure 3. Amplification of markers and genetic mapping of *TaPRR73-A1*: **A.** Amplification of functional marker PASF2/PASR2 in hexaploid materials: M: MarkerIII; 1: Yanzhan 1 (YZ1); 2: Neixiang 188; 3: Chinese Spring; 4: Lumai 14; 5: W7984; 6: AM3; **B.** Amplification of marker URSF1/URSR1 in diploid materials 1: UR203; 2: UR206; 3: A-B08; 4: A-BD104; 5: A-M0102; 6: A-M0101; **C.** Amplification of markers 73ASF1/73ASR1 in hexaploid and diploid materials 1:YZ1; 2: Neixiang 188; 3: Chinese Spring; 4: Lumai 14; 5: Fuzhuang 30; 6: Laizhou 953; 7: Hanxuan 10; 8: UR203; 9: UR206; 10: A-B08; 11: A-BD104; 12: A-M0102; **D.** *TaPRR73-A1* was mapped in chromosome 4A in a recombinant inbred line (RIL) population derived from the cross Yanzhan 1×Neixiang 188; **E.** Amplification of allele-specific markers in hexaploid materials; upper, CF4/R4, lower, TF4/R4. M: MarkerIII; 1: YZ1; 2: Neixiang 188; 3: Chinese Spring; 4: Lumai 14; 5: W7984; 6: AM3; 7: Fuzhuang 30; 8: Am6; 9: Opata; 10: Laizhou 953; 11: Hanxuan 10.

Supplementary sequence 1. The intact cDNA sequence of *TaPRR73-A1*, *TaPRR73-B1* and *TaPRR73-D1*, and the insert segment of T*aPRR73-B1* in transgenic rice. The overexpression sequence in transgenic rice is shown with a yellow background.

TaPRR73-A1 cDNA sequence:

ATGGTGAGCGCCGGTCAAGCTGGCGCGGACGGACCTTCCACCAGTGATATTAGGGGAACTGGAAACGGCGCCTTAGAGAA

TGGCCATGCCCTCAAGGCAAACGAGGACAAGGAATGGAGGGGCGGCAGCAAGGAAGAGGACTGGCCCAGCACGCACAGTG

CGCCGCCAGGCTTGGACGAGCACAAGCAGCAGCAAGGCCGGGTCATCCGCTGGGAGAAGTTCCTGCCGGTGGAGACACTA

AGGGTCTTGCTGGTGGAGAACGATGACTGTACCCGACATGTTGTCCGTGCTCTGCTCCGTAAGTGTGGCTATGAAGTTAT

CGCTGCTGAGAATGGATTGCATGCATGGCAATATCTTGAAGATGTGCAAAACCGTATTGACCTTGTATTAACTGAGGTCG

CCATGCCTTGTCTATCTGGAATTGGTCTACTCAGTAAGATCACGAGTCACAGTATTTGCAAGGGCATTCCTGTGATCATG

ATGTCTAAGAATGACTCGATGAGTACAGTCTTTAGGTGTCTATCAAAGGGAGCAGTTGACTTCTTAGTGAAGCCGATACG

GAAGAATGAACTTAAGACCCTTTGGCAGCATATATGGAGGCGATGCCACAGTTCCAGTGGAAGTGAAAGTGGCATCCATA

CACAAAAATGTTCCAAGCCGAGGGCTGGTGATGAATATGAGAACAACAGTGGCGGCAGTCATGATGATGATGACGATGAC

GATGATGATGCTGATGACGACTTTAGTGTTGGGCCCAATGCTAGGGATGGCAGTGATAATGGCAGTGGCACTCAGAGTTC

ATGGACGAAGCGTGCTGTGGAGATTGATAGCCCACAACTTGTGTCTTCTGATCATCTAGCAGATTCACCTGATAGTACTT

GTGCGCAAGTAATTCACCCCAGATCAGAGATAGGCAGCAATAGGTGGTTGCCGACTGCAAATAAAAGGAACATCAATAAT

CAAAAAGAAAATAATGATGACTCCATGGGGAAATACTTAGAAATAGGCGCTCCTAGAAATTCAAGTCTTGGGTATCAATC

TTCTCCAAATGAGATGTCTGTTAATCCAACAGAAAAACAGCATGAGAATCTCACATGTCAAAACAAGTCAGTAAACAAAA

TAGTCATCGACAAACCAACTAGTCAAACTGCTGATTTGATTAGTTCAATAGCCAGAAACACAGAATCGAAACAGGCTGCT

AGAATCACTGATGCACCTGACTGCTCCTCCAAGATGCCAAACGGGAATGAAATGAAAAACGATTCTCCCATCAACATGCC

ATCCCAAGAGCTGGGTCTGAAGATATCGGAAACAACTAGATGTGGAACTGAAATCCAGGATGAACGAAGTATTCTGAAAA

GATCAAATATCTCAGCATTCACCAGGTACCATACTCCCATGGCTTCCGATCAAGGTGGGGCAGCATTTCGGGGAAGCTGT

TCACCTCAAGATAACAGCTCAGAGGCTGTGAAAACGAACTCCACCTGCAAGATGGAGTCAAATTCAGATGCTGCTCAAAT

AAAGCAGGGCTCAAATGGCAGTAGCAACAACAATGACATGGGCTCCAGTACCAAGAATGCCATCGCAAAGCCTTGTACAG

ACAGGGAGAGAGTTATGTCACCATCACTTGTTAAATCAAACCAGCAGACTTCAGCATTCCATCCGATGCAGCACCGAGTG

TCACCAGCTGACGCAGCACGCAAGGACAAAGCTGCTGAAGAAATTGCCAATGCAGTGAAAGTGGGCCACTCAAGTGAGGC

ACAGCAAAGCTCTGTGCAGCATCACCATCATGCACACTATTACCGCCATGTTATGGCACAGCAACAGACATTAATTGACC

GTGCATCAAACGCCCGGTGTGGTTCATCCAATGCGTCTGATTTACCCCTGGAAGGTCATGCTGCCAACTATGGTGTGAAC

GGGAGCGTCTCAGGCAGCAATAATGGAAGCAATACGCAAAACGGAAGTAGCTCCGCTCCCAATATTGCAAGGCCAAACCT

GGACAGTGGTACCATGGACAAAACTGAGGCTGGCGGTGGCAACGGTAGTGGGAGCGGGCCGAGCGGTAGTGGCAATGACA

TGGTATGTCAGAATCAGCTCAGCCAACGAGAAGCTGCAGTGAACAAATTCAGGCAGAAGCGGAAAGAGAGGAACTTCGGG

AAAAAGGTGCGGTACCAAAGCAGGAAGAGACTTGCCGAGCAGCGGCCACGTGTCCGTGGGCAATTCGTCCGACAGTCTGG

ACAAGAAGATGACGCAGGCCAAGCAGCAGACAGATGA

TaPRR73-B1 cDNA sequence:

ATGGTGAGCGCCGGTCAAGCTGGCGCGGACGGACCTTCCACCAGTGATATTAGGGGAACTGGAAACGGCGCCGTAGAGAATGGCCATGCCCTCAAGGCAAACGAGGACAAGGAATGGAGGGGCGGCAGCAAGGAAGAGGACTGGCCCAGCACGCACAGTGCGCCGCCAGGCTTGGACGAGCACAAGCAGCAGCAAGGCCGGGTCATCCGCTGGGAGAAGTTCCTGCCGGTGGAGACACTAAGGGTCTTGCTGGTGGAGAACGATGACTGTACCCGACATGTTGTCCGTGCTCTGCTCCGTAAGTGTGGCTATGAAGTTATCGCTGCTGAGAATGGATTGCATGCATGGCATTATCTTGAAGATGTGCAAAACCGTATTGACCTTGTATTAACTGAGGTCGCCATGCCTTGTCTATCTGGCATCGGTCTACTCAGTAAGATCACGAGTCACAGTATTTGCAAGGGCATTCCTGTGATCATGATGTCTAAGAATGACTCGATGAGTACAGTCTTTAAGTGTCTATCAAAGGGAGCAGTTGACTTCTTAGTGAAGCCGATACGGAAGAATGAACTTAAGACCCTTTGGCAGCACATATGGAGGCGATGCCACAGTTCCAGTGGAAGTGAAAGTGGCATCCATACACAAAAATGTTCCAAACCAAAGGCTGGTGATGAATATGAGAACAACAGTGGCGGCAGTCATGATGATGATGACGATGACGATGATGATGCTGACGACGACTTTAGTGTTGGGCCCAATGCTAGGGATGGCAGTGATAATGGCAGTGGCACTCAGAGTTCATGGACGAAGCGTGCTGTGGAGATTGATAGTCCACAACTTGTGTCTTCTGATCATCTAGCAGATTCACCTGATAGTACCTGTGCGCAAGTAATTCACCCCAGATCAGAGATAGGCAGCAATAGGTGGTTGCCGACTGCAAATAAAAGGAACATCAATAATCAAAAAGAAAATAATGATGACTCCATGGGGAAATACTTAGAAATAGGCGCTCCTAGAAATTCAAGTCTTGGGCATCAATCTTCTCCAAATCAGATGTCTGTTAATCCAACAGAAAAACAGCATGAGAATCTCATATCCCAAAACAAGTCCGTAAACAAAATAGTCATCGACGAACCAACTAGTCAAACTGCCGATTTGATTAGTTCAATAGCCAGAAACACAGAATCGAAACAGGCTGCTAGAATCACTGATGCACCTGATTGCTCCTCCAAGATGGCACACGGGACTGAAATGAAAAACGATTCTCCCATCAACATGCCATCCCAAGAGTTGGGTCTGAAGATATCGGAAACAGCTAGATGTGGAACTGAAATCCATGATGAACGAAGTATTCTGAAAAGATCAAATCTCTCAGCATTCMCCAGGTACCATACTCCTATGGCTTCCGATCAAGGTGGGGCAACATTTCGGGGAAGCTGTTCACCTCAAGATAACAGCTCAGAGGCTGTGAAAACGAACTCCACCTGCAAGATGGAGTCAAATTCAGATGCTGCTCAAATAAAGCAGGGCTCAAATGGCAGTAGCAACAACAATGACATGGGCTCCAGTACCAAGAATGCCAtTGCAAAGCCTTGTACAGACAGGGAGAGAGTTATGTCACCATCACTTGTTAAATCGAACCAGCAGACTTCAGCATTCCATCCGGTGCAGCACCAAGTGTCACCAGCTGACGCGGCACGCAAGGACAAAGCTTCTGAAGAAATTGTCAATGCAGTGAAAGTGGGCCACTCAAGCGAGGCACAGCAAAGCTCTGTGCAGCATCACCATCATGCACACTATTACCGCCATGTTATTGCACAGCAACAGACATTAATTGACCGTGCATCAAACGCTCGGTGTGGTTCATCCAATGCGTCTGATTTACCCCTGGAAGGTCATGCTGCTAACTATGGTGTGAACGGGAGCATCTCAGGCAGCAATAATGGAAGCAATACGCAAAACGCAAGTAGCTCCGCTCCCAATATTGCAAGGCCAAACATGGACAGTGGTGCCATGGACAAAACAGAGGCTGGCGGTGGCACCGGTAGTGGGAGCGGGCCGAGCGGTAGTGGCAATGACATGGTATGTCAGAATCAGCTCAGCCAACGAGAAGCTGCAGTGAACAAATTCAGGCAGAAGCGGAAAGAGAGGAACTTCGGGAAAAAGGTGCGGTACCAGAGCAGGAAGAGACTAGCCGAGCAGCGGCCACGTGTCCGTGGGCAATTCGTCCGACAGTCTGGACAAGAAGATGAGGCAGGCCAAGCAGAAGACAGATGA

TaPRR73-D1 cDNA sequence:

ATGGTGAGCGCCGGTCAAGCTGGCGCGGACGGACCTTCCACCAGTGATATTAGGGGAACCGGAAACGGCGCCGTAGAGAA

TGGCCATGCCCTCGAGGCAAACGAGGACAAGGAATGGAGGGGCGGCATCAAGGAAGAGGACTGGCCCAGCACGCACAGTG

CGCCGCCGGGCTTGGACGAGCAGAAGCAGCAGCAAGACCGGGTTATCCGGTGGGAGAAGTTCCTGCCGGTGAAGACACTA

AGGGTCTTGCTGGTGGAGAACGATGACTGTACCCGACATGTTGTCCGTGCTCTGCTCCGTAAGTGTGGCTATGAAGTTAT

CTCTGCTGAGAATGGATTGCATGCATGGCAATATCTTGAAGATGTGCAAAACCGTATTGACCTGGTATTAACCGAGGTCG

CCATGCCTTGTCTATCTGGCATTGGTCTGCTCAGTAAGATCACGAGTCGCAGTATTTGCAAGGGCATTCCTGTGATCATG

ATGTCTAAGAATGACTCGATGAGTACAGTCTTTAGGTGTCTATCAAAGGGAGCAGTTGACTTCTTAGTGAAGCCGATACG

GAAGAATGAACTTAAGACCCTTTGGCAGCACATATGGAGGCGATGCCACAGTTCCAGTGGAAGTGAAAGTGGCATCCATA

CACAAAAATGTTCCAAACCGAAGGCTGGTGATGAATATGAGAACAACAGTCATGATGACGATGACGATTGCGGCAGTCAT

GATGACGATGACGATGACGATGATGATGCCGATGACGACTTTAGTGTTGGGCCCAATGCTAGGGATGGCAGTGATAATGG

CAGTGGCACTCAGAGTTCATGGACGAAGCGTGCTGTGGAGATTGATAGCCCACAACTTTTGTCTTCTGATCATCTAGCAG

AATCACCTGATAGTACTTGTGCGCAAGTAATTCACCCCAGATCAGAGATAGGCAGCAATAGGTGGTTGCCGACTGCAAAT

AAAAGGAACATCAATAATCAAAAAGAAAATAATGATGACTCCATGGGGAAATACTTAGAAATAGGCGCTCCTAGAAATTC

AAGTCTTGGGTATCAATCTTCTCCAAATGAGACGTCTGTTAATCCAACAGAAAAACAGCATGAGAATCTCACATCCCAAA

ACAAGTCAGTAAACAAAATAGTCATCGACAAACCAACTTGTCAAACTGCTGATTTGATTAGTTCAATAGCCAGAAACACA

GAATCGAAACAGGCTGCTAGAATCACTGATGCACCTGACTGCTCCTCCAAGATGCCAAACGGGAATGAAATGAAAAACGA

TTCTCCCATCAACATGCCATCCCAAGAGCTGGGTCTGAAGATATCGGAAACAACTAGATGTGAAACTGAAATCCATGATG

AACGAAGTATTCCGAAAAGATCAAATCTGTCAGCATTCACCAGGTACCATACTCCCATGGCTTCCGATCAAGGTGGGGCA

ACATTTCGGGGAAGCTGTTCACCTCAAGATAACAGCTCAGAGGCTGTGAAAACGAACTCCACCTGCAAGATGGAGTCAAA

TTCAGATGCTGCTCAAATAAAGCAGGGCTCAAATGGCAGTAGCAACAACAATGACATGGGCTCCAGTACAAAGAGTGCCA

TCGCAAAGCCTTGTACAGACAGGGAGAGAGTTATGTTACCATCACTTGTTAAATCGAACCAGCAGACTTCAGCATTCCAT

CCGGTGCAGCACCAAGTGTCACCAGCTCATGTGGCACGCAAGGACAAAGCTGCTGAAGAAATTGCCAATGCAGTGAAAGT

GGGCCACTCAAGCGAGGCACAGCAAAGCTCTGTGCAGCATCACCATCATGCACACTATTACCGCCATGTTATGGCACAGC

AACAGACATTAATTGACGGTGCATCAAACGCTCGGTGTGGCTCATCCAATGCTTCCGATTCACCCATGGAAGGTCATGCT

GCTAACTATGGTGTGAATGTGAGCGTCTCAGGCAGCAATAATGCAAGCAATACGCAGAATGGAAGTAGCTCCGCTCCCAA

TATTGCAAGGCCAAACATGGAGAGTGGTACCATGGACAAAATTGAGGCTGACGGTGGCAATGGCAGCGGGAGCAGGCCGA

GCGGTAGTGGCAATGACATGGTTTGTCAGAATCAGCTCAGCCAACGAGAAGCTGCAGTGAACAAATTCGGGCAGAAGCGG

AAAGAGAGGAACTTCGGGAAAAAGGTGCGGTACCAAAGCAGGAAGAGACTGGCCGAGCAGCGGCCACGTGTCCGTGGGCA

ATTCGTTCGACAGTCTGGACAAGATGAGGCAGGCCAAGCAGAAGACGGATGA

PR domain

The amino acid sequence of TaPRR73-B1:

MVSAGQAGADGPSTSDIRGTGNGAVENGHALKANEDKEWRGGSKEEDWPSTHSAPPGLDEHKQQQGRVIRWEKFLPVETLRVLLVENDDCTRHVVRALLRKCGYEVIAAENGLHAWHYLEDVQNRIDLVLTEVAMPCLSGIGLLSKITSHSICKGIPVIMMSKNDSMSTVFKCLSKGAVDFLVKPIRKNELKTLWQHIWRRCHSSSGSESGIHTQKCSKPKAGDEYENNSGGSHDDDDDDDDDADDDFSVGPNARDGSDNGSGTQSSWTKRAVEIDSPQLVSSDHLADSPDSTCAQVIHPRSEIGSNRWLPTANKRNINNQKENNDDSMGKYLEIGAPRNSSLGHQSSPNQMSVNPTEKQHENLISQNKSVNKIVIDEPTSQTADLISSIARNTESKQAARITDAPDCSSKMAHGTEMKNDSPINMPSQELGLKISETARCGTEIHDERSILKRSNLSAFXRYHTPMASDQGGATFRGSCSPQDNSSEAVKTNSTCKMESNSDAAQIKQGSNGSSNNNDMGSSTKNAIAKPCTDRERVMSPSLVKSNQQTSAFHPVQHQVSPADAARKDKASEEIVNAVKVGHSSEAQQSSVQHHHHAHYYRHVIAQQQTLIDRASNARCGSSNASDLPLEGHAANYGVNGSISGSNNGSNTQNASSSAPNIARPNMDSGAMDKTEAGGGTGSGSGPSGSGNDMVCQNQLSQREAAVNKFRQKRKERNFGKKVRYQSRKRLAEQRPRVRGQFVRQSGQED

CCT motif

**References to Supplementary files**

Schmid, M., Davison, T.S., Henz, S.R., Pape, U.J., Demar, M., Vingron, M., et al. (2005) A gene expression map of Arabidopsis thaliana development. *Nat Genet.* 37: 501-506.

Zimmermann, P., Hirsch-Hoffmann, M., Hennig, L., Gruissem, W. (2004) GENEVESTIGATOR. Arabidopsis microarray database and analysis toolbox. Plant Physiology. 136: 2621-2632.
